# Percutaneous Auricular Vagus Nerve Stimulation Reduces Inflammation in Critical Covid-19 Patients

**DOI:** 10.3389/fphys.2022.897257

**Published:** 2022-07-04

**Authors:** Tamara Seitz, József Constantin Szeles, Reinhard Kitzberger, Johannes Holbik, Alexander Grieb, Hermann Wolf, Hüseyin Akyaman, Felix Lucny, Alexander Tychera, Stephanie Neuhold, Alexander Zoufaly, Christoph Wenisch, Eugenijus Kaniusas

**Affiliations:** ^1^ Department of Infectious Diseases and Tropical Medicine, Klinik Favoriten, Vienna, Austria; ^2^ Department of General Surgery, Division of Vascular Surgery, Medical University of Vienna Center for Wound Surgery, Health Service Center of Vienna Privat Clinics, Vienna, Austria; ^3^ Immunology Outpatient Clinic, Vienna, Austria; ^4^ Medical School, Sigmund Freud Private University, Vienna, Austria; ^5^ Faculty of Electrical Engineering and Information Technology, Institute of Biomedical Electronics, Vienna University of Technology (TU Wien), Vienna, Austria

**Keywords:** COVID-19, SARS-CoV-2, hyperinflammation, cytokine storm, aVNS, nervus vagus stimulation

## Abstract

Covid-19 is an infectious disease associated with cytokine storms and derailed sympatho-vagal balance leading to respiratory distress, hypoxemia and cardiovascular damage. We applied the auricular vagus nerve stimulation to modulate the parasympathetic nervous system, activate the associated anti-inflammatory pathways, and reestablish the abnormal sympatho-vagal balance. aVNS is performed percutaneously using miniature needle electrodes in ear regions innervated by the auricular vagus nerve. In terms of a randomized prospective study, chronic aVNS is started in critical, but not yet ventilated Covid-19 patients during their stay at the intensive care unit. The results show decreased pro-inflammatory parameters, e.g. a reduction of CRP levels by 32% after 1 day of aVNS and 80% over 7 days (from the mean 151.9 mg/dl to 31.5 mg/dl) or similarly a reduction of TNFalpha levels by 58.1% over 7 days (from a mean 19.3 pg/ml to 8.1 pg/ml) and coagulation parameters, e.g. reduction of DDIMER levels by 66% over 7 days (from a mean 4.5 μg/ml to 1.5 μg/ml) and increased anti-inflammatory parameters, e.g. an increase of IL-10 levels by 66% over 7 days (from the mean 2.7 pg/ml to 7 pg/ml) over the aVNS duration without collateral effects. aVNS proved to be a safe clinical procedure and could effectively supplement treatment of critical Covid-19 patients while preventing devastating over-inflammation.

## Introduction

### Covid-19

Covid-19 is an infectious disease caused by SARS-CoV-2 invading epithelial cells via angiotensin 2-converting enzyme (ACE2) receptors abundantly expressed in alveolar epithelial cells as well as in the heart (Hoffmann et al., 2020). Covid-19 may lead to severe pro-inflammatory cytokine storms and hemophagocytosis, derailed sympatho-vagal balance, culminating in severe hypoxemia, major respiratory distress, cardiovascular damage, and increased thrombotic and/or thromboembolic events. In particular, elevated levels of interleukin-1β, IL-2, IL-6, IL-10, TNF, IFNalpha, IFNbeta, interferon-γ, macrophage inflammatory proteins 1α and 1β, and VEGF appear in patients with Covid-19–associated cytokine storm (Huang et al., 2020; Yi et al., 2020; Zhu et al., 2020). Several clinical and laboratory abnormalities, such as elevated CRP, ferritin and DDIMER levels are described during cytokine storm in addition to the elevated systemic cytokine levels (Fajgenbaum and June 2020). Further, on the one hand a repression of anti-inflammatory cytokines, such as IL-10, on the other hand an excessive secretion was described in critical COVID-19 (Rabaan et al., 2021).

### The Vagally-Controlled Immune System

The immune system is mediated and modulated by afferent and efferent fibers of the vagus nerve, the major nerve of the parasympathetic nervous system (Pavlov and Tracey, 2012). The nerve acts as a major component of the neuroendocrine-immune axis (Bonaz, Sinniger and Pellissier, 2016) and is a key factor in response to infection. While the parasympathetic vagus nerve exerts only anti-inflammatory effects, the sympathetic nervous system may have both pro-inflammatory and anti-inflammatory effects. The vagus nerve is involved in multiple pathways within immune reflexes: the anti-inflammatory hypothalamic-pituitary-adrenal axis (HPAA) and the cholinergic anti-inflammatory pathway (ChAIP) (Borovikova et al., 2000; Tracey, 2007; Bonaz, Sinniger and Pellissier, 2016).

In both reflexes, inflammatory mediators (e.g., pro-inflammatory cytokines and/or endotoxins) activate the afferent vagal fibers projecting this inflammatory information to the nucleus of the solitary tract (NST) which, in turn, project to the dorsal motor nucleus of the vagus nerve. In the HPAA, activated efferents to hypothalamus stimulate the release of corticotrophin-releasing hormone which stimulates the secretion of adrenocorticotropic hormone from the pituitary gland. The adrenocorticotropic hormone reaches adrenal glands and stimulates the release of glucocorticoids, acting on the spleen so that pro-inflammatory cytokines and the resulting peripheral inflammation are reduced. In the ChAIP, activated cholinergic efferents to the spleen release acetylcholine at their preganglionic synaptic endings, with acetylcholine binding to surface receptors of macrophages and suppressing the release of pro-inflammatory cytokines by these macrophages.

In view of cytokine storms in Covid-19, it is instructive to observe that over-inflammation with the associated unrestrained cytokine release results when the tonic neural activity of ChAIP is impaired (Mercante, Deriu and Rangon, 2018), which highlights the importance of ChAIP, the involved vagus nerve and its stimulation.

### The Vagus Stimulation

Vagus nerve stimulation can be performed through invasive, non-invasive, and minimally-invasive methods (Kaniusas et al., 2019a). For invasive stimulation, a cuff electrode is implanted at the cervical level typically wrapped around the left cervical branch of the vagus nerve, showing high implantation risks and costs (Mertens et al., 2018). For non-invasive stimulation, surface skin electrodes are used on the outer ear (Ellrich, 2011), yielding easy-to-use, but diffuse transcutaneous stimulation of both vagally and non-vagally innervated regions. The associated minor side effects include headache, pain and skin irritation at the stimulation site. The afferent branches of the cervical vagus nerve can also be non-invasively stimulated via surface skin electrodes of a hand-held device applied at the neck (Barbanti et al., 2015), with potential side effects such as prickling at stimulation site, neck pain, dizziness, headache.

We focus on the percutaneous minimally invasive auricular vagus nerve stimulation (aVNS) where miniature needle electrodes are positioned in the outer ear regions innervated mainly by the vagus nerve (Kaniusas and Samoudi, 2020). The percutaneous aVNS shows minor side effects (Kaniusas et al., 2019a; Kaniusas, et al., 2019b): local skin irritation of the ear (with the incidence <10%) and inadvertent bleeding (<1%) can occur, which can be reduced down to <0.05% when erroneous placement of needles directly into auricular vessels is avoided using transillumination of the outer ear to visualize auricular vascularization (Kampusch et al., 2016; Roberts et al., 2016).

The percutaneous aVNS targets Aβ-fibers in the ear responsible for cutaneous mechanoreception and touch sensation, which project to NST in the brainstem and activate visceral and somatic projections (Frangos, Ellrich and Komisaruk, 2015). The NTS is involved in the aforementioned control of autonomic immune systems, as well as cardiorespiratory and cardiovascular regulation (Thayer, Mather and Koenig, 2021).

### The Auricular Vagus Stimulation in Immune System

aVNS reduced pro-inflammatory cytokines in atrial fibrillation (Stavrakis et al., 2015) and increased norepinephrine levels (Beekwilder and Beems, 2010) which supports anti-inflammatory aVNS effects. aVNS reduced systemic tumor necrosis factor in mice with lethal endotoxemia or polymicrobial sepsis (Huston et al., 2007). aVNS reduced pro-inflammatory cytokines and suppressed lipopolysaccharide-induced inflammatory responses in endotoxemic rats (Zhao et al., 2012). In humans, anti-inflammatory effects were shown in rheumatoid arthritis (Bernateck et al., 2008; Koopman et al., 2016), inflammatory bowel disease (Crohn’s disease, ulcerative colitis), and postoperative ileus (Tracey, 2007; Marshall et al., 2015; Bonaz, Sinniger and Pellissier, 2016), and lobectomy (Salama, Akan and Mueller, 2017).

### Hypothesis

In line with our theoretical hypothesis (Kaniusas and Szeles, 2020a) suggesting aVNS as a potential treatment of Covid19-originated acute respiratory distress syndrome and the associated co-morbidities through activation of anti-inflammatory pathways, we present here a randomised prospective study on clinical effects of aVNS on inflammation related markers in critical but not yet ventilated patients.

## Methods

### Patient Recruitment

Patients admitted to the intensive care unit (ICU) of the Department of Infectious Diseases and Tropical Medicine, Klinik Favoriten, Vienna, Austria due to Covid-19 were screened by the attending physician regarding the following inclusion and exclusion criteria:

Inclusion criteria (all criterion are needed for inclusion):

• Positive for SARS-CoV-2 by RT-PCR test (defined as a C_T_ value less than 30)
• Acute respiratory failure requiring non-invasive respiratory support
• PaO_2_/FiO_2_ <200

Exclusion criteria (one criteria is sufficient for exclusion):

• Age <18 years
• Pregnancy (to be excluded using serum beta HCG in women of childbearing age)
• Signs of infection, eczema, or psoriasis at the application site
• Active malignancy
• Implanted cardiac pacemaker, defibrillator, or other active implanted electronic devices
• Patient unable to consent
• Heart rate <60 beats/min
• Known vagal hypersensitivity
• History of haemophilia

If all inclusion criteria and none of the exclusion criteria were met, the attending physician informed the patient about the study. After written consent was obtained, randomisation of the study group (*n* = 10) into the VNS group or the standard of care (SOC) group was performed with a computer-based randomisation tool. The time of inclusion was considered as the time point 0 (T0). Following the COVID-19 guidelines of Open Critical Care (COVID-19 Guidelines Dashboard - Open Critical Care, 2021), SOC implicates that patients with respiratory insufficiency receive 10 mg of dexamethasone for 10 days and prophylactic anticoagulation therapy.

### Procedure

aVNS was started immediately for patients selected in the VNS group. This procedure was performed using an AuriStim device (Multisana GmbH, Austria). AuriStim is a single-use, miniaturized, and battery-powered electrical stimulator. The stimulator delivers monophasic varying polarity pulses (pulse width 1 ms) with a fixed amplitude (3.8 V) every second (stimulation frequency is 1 Hz), and a duty cycle (3 h ON/3 h OFF). The resistance of the needle and needle to tissue interface is about 4–7kOhm so that the resulting peak current amount to about 0.5–0.9 mA, residing at or below the suggested limits of about 1.5 mA.

Multi-punctual percutaneous aVNS was mediated via three miniature needle electrodes inserted into vagally (solely or partly) innervated regions of the auricle. These regions were the cymba concha (vagal nerve is found in 100% of cases) (Peuker and Filler, 2002), cavity of concha (45%), and the crura of antihelix (9%). Needles were located close to local blood vessels—as identified by transillumination of the auricle (Kaniusas et al., 2011)—with the auricular nerves nearby (Dabiri et al., 2020).

Following insertion of the needles, an intermittent stimulation cycle of 3 hours of activity and 3 hours of rest was initiated, equating to four cycles of 3 hours of stimulation in 24 h. During the treatment period the device was swapped to avoid a decrease of function due to low battery. The procedure was performed until either the patient was discharged from the ICU, transferred to another ward, or died.

If patients were responsive, the visual analogue scale (VAS) was documented four times per day and at time of device placement to document pain at the insertion site. The VAS is a Likert scale that ranges from 0 (“no pain”) to 10 (“pain as bad as it could possibly be”). If the VAS was over five during aVNS, the stimulation was stopped. Patients had the option of terminating the procedure immediately if they wished due to discomfort. Furthermore, patients were clinically examined and interviewed for potential side effects of aVNS.

To evaluate inflammation, macrophage activation, anti-inflammatory, and coagulation biomarkers, blood samples (1 serum tube per time point) were drawn in both groups at the following time points:

• Day 1 (every 4 h): T0 (Time of inclusion in the study), T4 (4 h after T0), T8, T12, T16
• Day 2 (every 8 h): T24, T32, T40
• Day 3 (every 12 h): T48, T60
• Day 4 (every 12 h): T72, T84
• Day 5 (every 12 h): T96, T108
• Day 6 (every 12 h): T120, T132
• Day 7 (every 12 h): T144, T156
• The blood samples were immediately centrifuged for 15 min at 3,000 rpm and then serum was frozen at −20°C. The analysis was performed within a 2-week period at a certified immunology laboratory.
• Supplementary blood samples were drawn every day between 6:00 and 6:30 a.m. for additional inflammation (CRP, ferritin) and coagulation (DDIMER, fibrinogen) biomarkers that were tested immediately afterwards.

Blood samples were collected for 7 days after study inclusion or until patients were discharged from this ICU, transferred to another ward, or died.

### Statistical Analysis

Basic characteristics of the participants were collected including gender, virus variant, time since symptom onset, comorbidities, and respiratory situation. The study presents a Proof of Concept- Study and financial resources were limited, therefore no power analysis was performed prior to the study.

The mean, median, standard deviation, minimum, and maximum were calculated for categorical variables.

Inflammation parameters were analysed on the log-scale using linear mixed models. A random intercept was introduced for each patient and a time-dependent correlation structure was modelled for the residual variance-covariance matrix, taking into account the unequally spaced intervals. A heteroscedasticity correction was applied when residuals were not homogeneously distributed over time. Restricted maximum likelihood was used to estimate the model parameters. A type I error rate of 5% was used for inferential statistics. In each model, we checked that the residuals were homoskedastic. The analyses were performed with R Statistics 4.1.1.

### Ethical Considerations

The study was approved by the local ethics committee (EK 21-079-0521) and Austrian Federal Office for Safety in Health Care BASG. The study was registered at ClinicalTrials.gov (NCT05058742).

## Results

The study was conducted from June to December 2021 and included 10 patients (5 patients randomised each to the VNS and SOC groups). Basic characteristics of the study participants are listed in Table 1. The mean age of the participants was 53–56 years, in the majority the virus variant B.1.617.2 was detected.

**TABLE 1:** Basis parameters of the study participants.

	VNS (*n* = 5)	SOC (*n* = 5)
Age (in years)		
Mean	55.6	53.2
Min-Max	42–68	43–64
Standard deviation	8.69	7.83
Gender		
	40% female	60% female
	60% male	40% male
Comorbidities		
Hypertension	60%	40%
Obesity	80%	80%
Mean BMI	35.32	33.22
Diabetes	40%	60%
Chronic artery disease	20%	0%
Chronic renal failure	20%	0%
Chronic lung disease	0%	0%
Thyroid disease	0%	20%
Active cancer	0%	0%
Hematological Disease	0%	0%
Rheumatological disease	0%	20%
Current Smoking	20%	0%
SARS-CoV-2 Vaccination	0%	0%
Virus variant	80% B.1.617.2 (Delta) 20% unknown	100% B.1.617.2 (Delta)
Time between symptom onset and ICU admission (in days)		
Mean	9.60	7.80
Min-Max	7–16	5–12
Standard deviation	3.26	2.64
Time between symptom onset and study Inclusion (in days)		
Mean	10.4	8.4
Min-Max	8–17	6–12
Standard deviation	3.32	2.06
Need of non-invasive ventilation at time of study inclusion	80%	80%
Horowitz Index at time of study inclusion		
Mean	124.7	103.8
Min-Max	69.8–190.7	65.6–180
Standard deviation	46.95	40.81
Length of aVNS (in days)		
Mean	12	—
Min-Max	3–18	—
Standard deviation	6.23	—
Therapy		
Corticosteroids	100%	100%
Other immunosuppressive agents	0%	0%
Antimicrobial therapy	80%	80%

### Tolerance and Safety

No adverse event was documented while using aVNS. No patient opted to terminate aVNS early. Mean VAS at the time of device placement was 3.4 (range 2–5). Otherwise, VAS was documented four times a day. The mean VAS was 1.9 (range 0–3). Eighty percent required non-invasive ventilation at the time of study inclusion with a Horrowitz Index between 65.6—190.7.

### Inflammation Parameters

Serum levels of IL-6, IFNγ, TNFα, Calprotectin, IL-18, S100A12, sIL2-receptor and IL-10 were analysed at an average of 16.1 time points (range 9–20) and a mean time of 6 days. CRP, ferritin, Fibrinogen and DDIMER levels at an average of six time points (range 2–8) and a mean time of 6 days per patient.

The inflammatory parameter levels at the time points 4, 24, 72, and 168 h in patients of the VNS group compared to the SOC group are shown in Figures 1–11. IFNγ did not show an elevated value at any timepoint.

**FIGURE 1:**
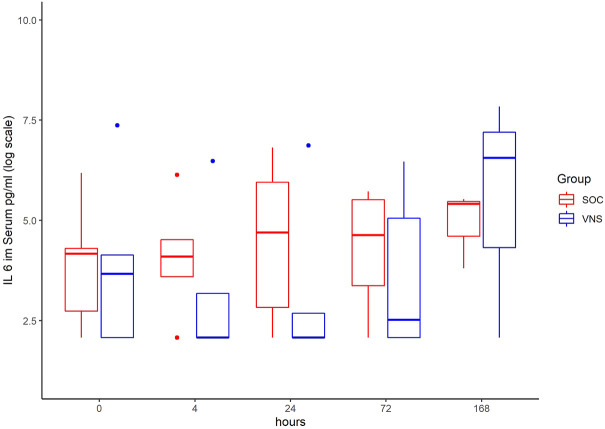
Median IL-6 level with Interquartile Range of patients receiving auricular Vagus Stimulation (VNS = blue) and patients only receiving Standard of Care (SOC = red) after 0, 4, 24, 72, and 168 h of study inclusion.

**FIGURE 2:**
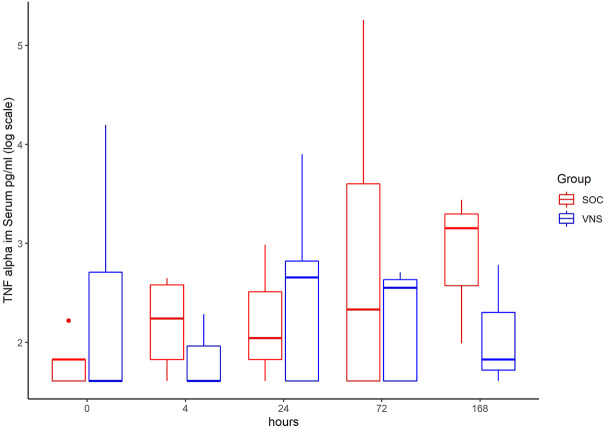
Median TNF alpha level with Interquartile Range of patients receiving auricular Vagus Stimulation (VNS = blue) and patients only receiving Standard of Care (SOC = red) after 0, 4, 24, 72, and 168 h of study inclusion.

**FIGURE 3:**
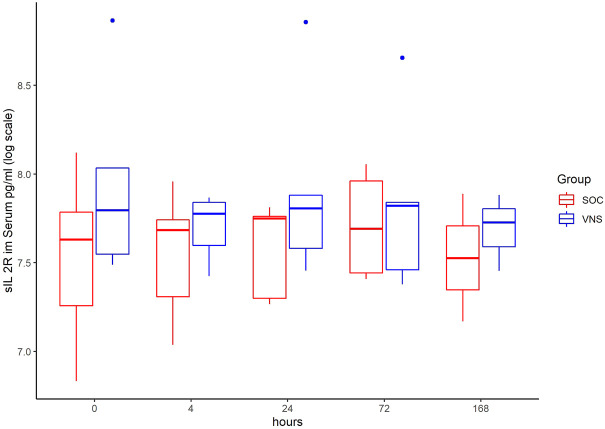
Median sIL-2R level with Interquartile Range of patients receiving auricular Vagus Stimulation (VNS = blue) and patients only receiving Standard of Care (SOC = red) after 0, 4, 24, 72, and 168 h of study inclusion.

**FIGURE 4:**
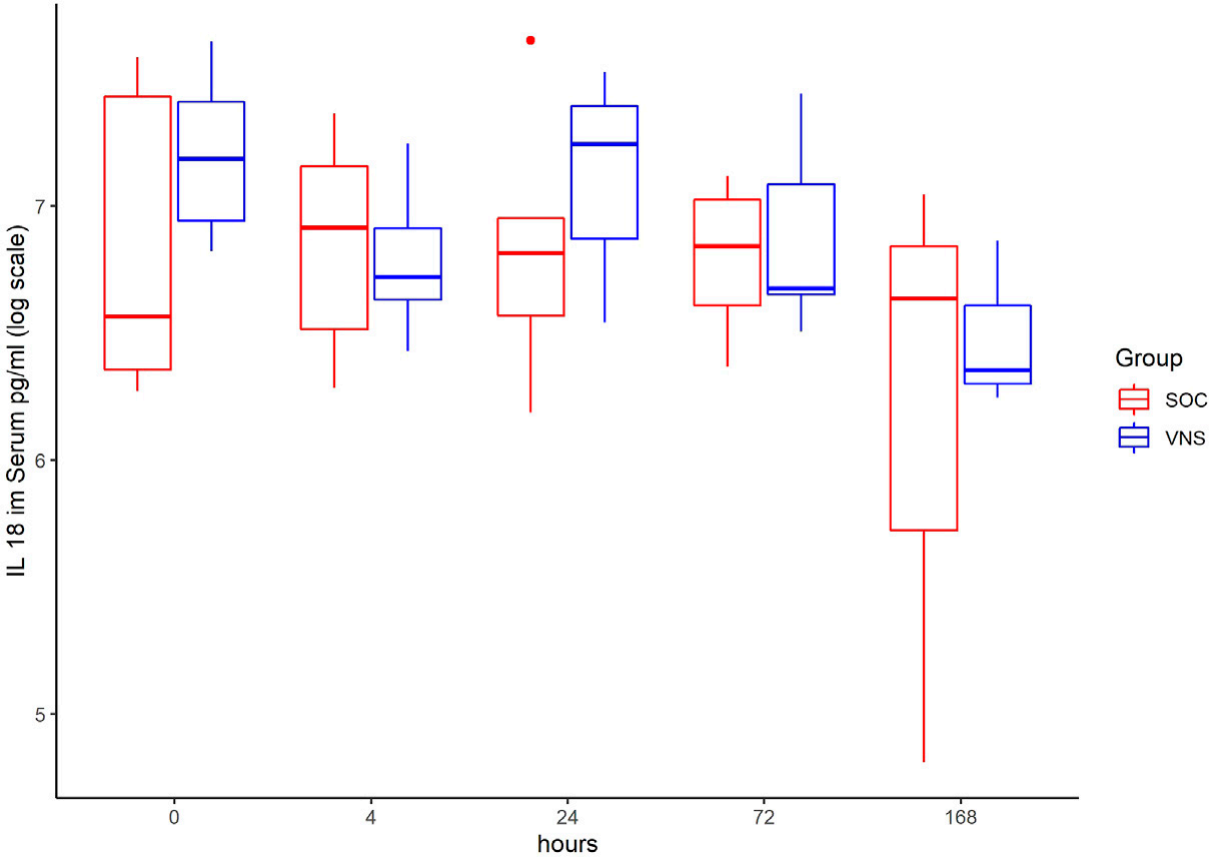
Median IL-18 level with Interquartile Range of patients receiving auricular Vagus Stimulation (VNS = blue) and patients only receiving Standard of Care (SOC = red) after 0, 4, 24, 72, and 168 h of study inclusion.

**FIGURE 5:**
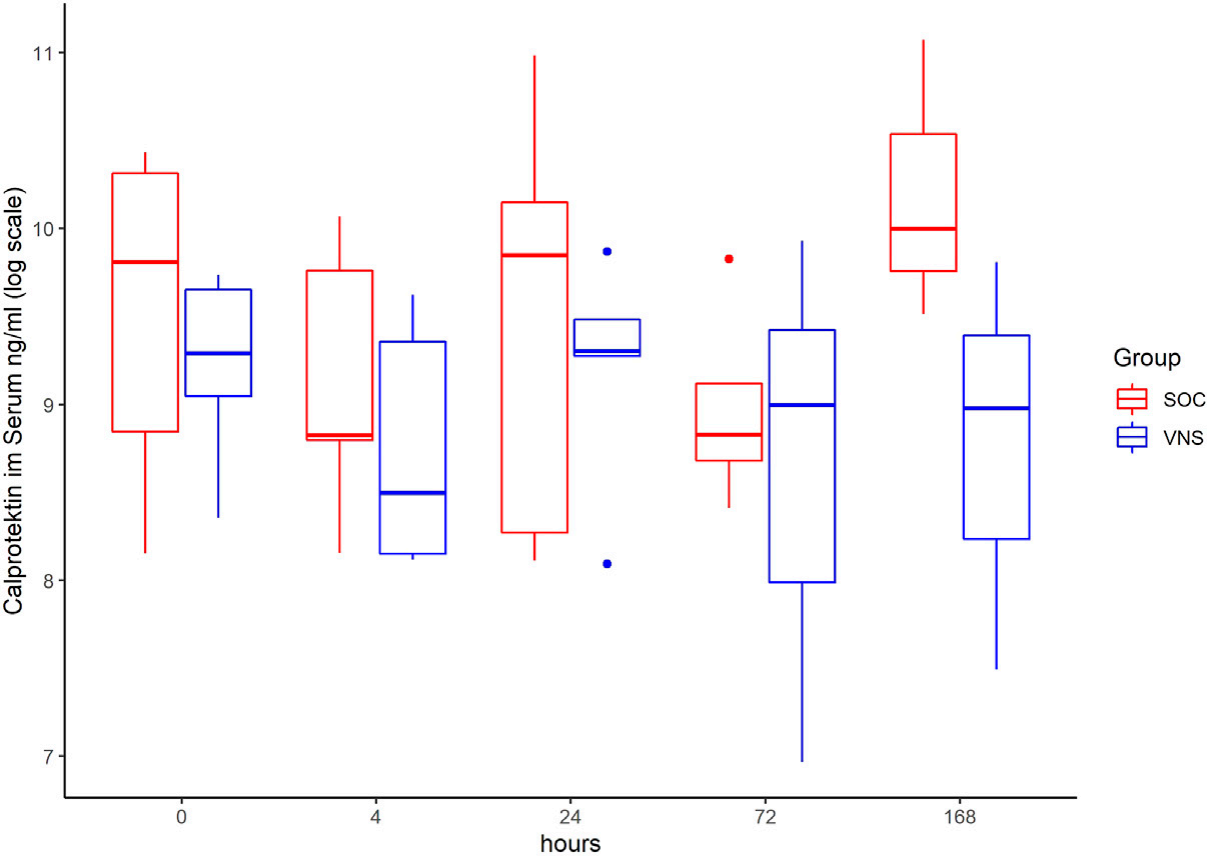
Median Calprotektin level with Interquartile Range of patients receiving auricular Vagus Stimulation (VNS = blue) and patients only receiving Standard of Care (SOC = red) after 0, 4, 24, 72, and 168 h of study inclusion.

**FIGURE 6:**
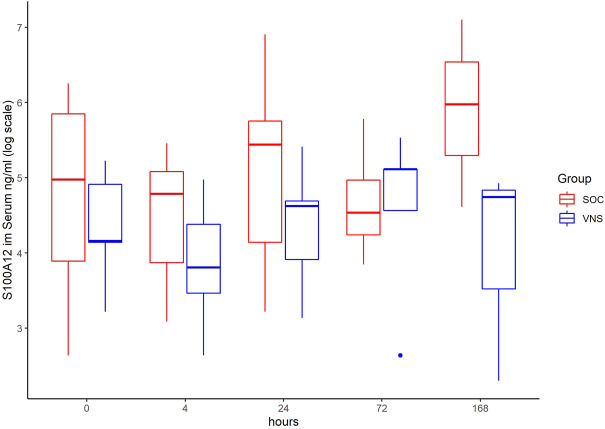
Median S100A12 level with Interquartile Range of patients receiving auricular Vagus Stimulation (VNS = blue) and patients only receiving Standard of Care (SOC = red) after 0, 4, 24, 72, and 168 h of study inclusion.

**FIGURE 7:**
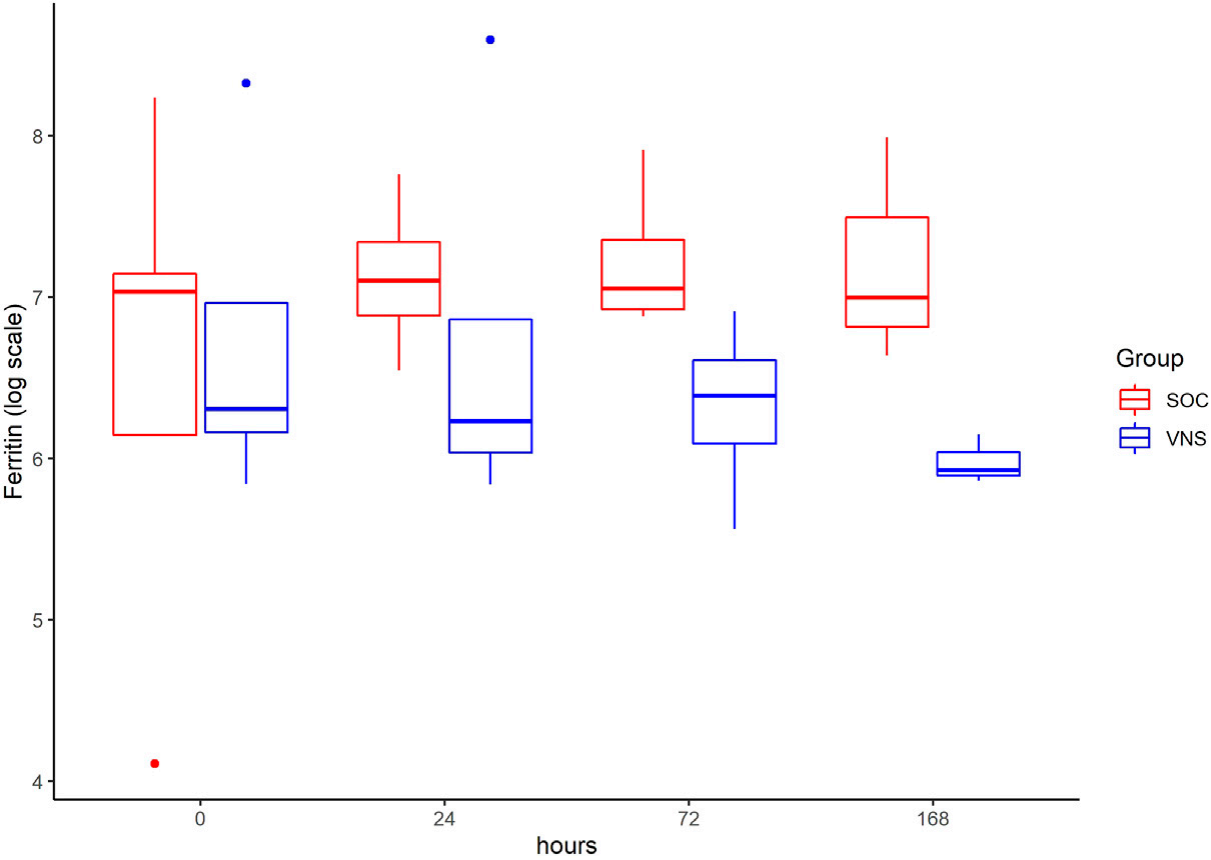
Median ferritin level with Interquartile Range of patients receiving auricular Vagus Stimulation (VNS = blue) and patients only receiving Standard of Care (SOC = red) after 0, 24, 72, and 168 h of study inclusion.

**FIGURE 8:**
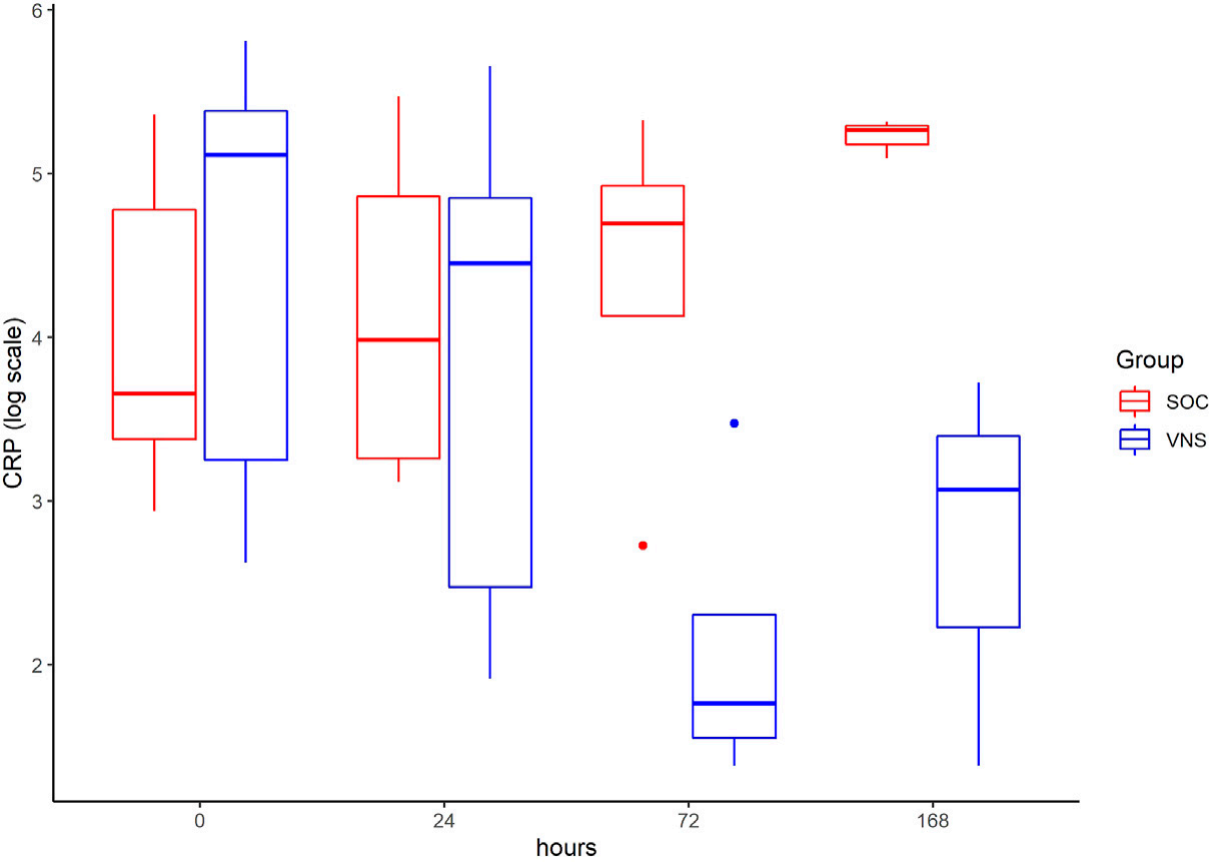
Median CRP level with Interquartile Range of patients receiving auricular Vagus Stimulation (VNS = blue) and patients only receiving Standard of Care (SOC = red) after 0, 24, 72, and 168 h of study inclusion.

**FIGURE 9:**
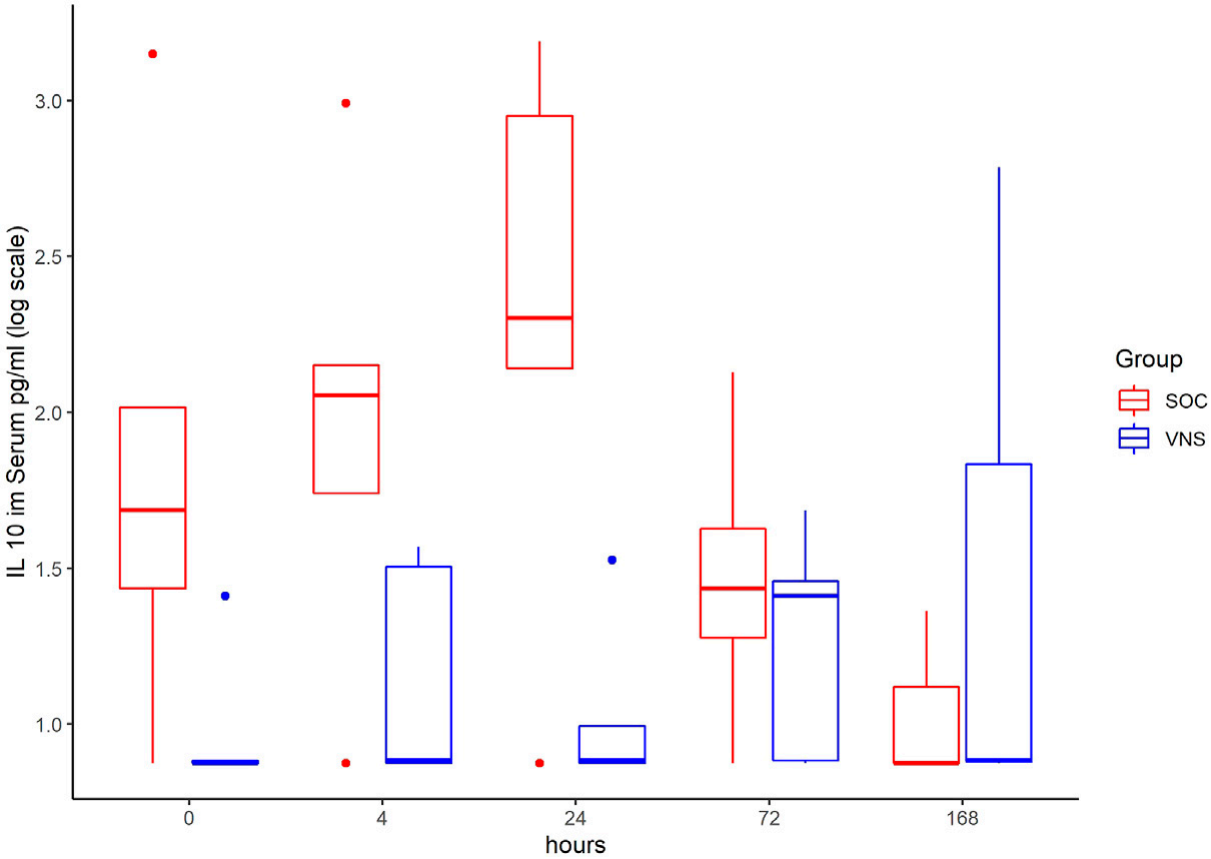
Median IL-10 level with Interquartile Range of patients receiving auricular Vagus Stimulation (VNS = blue) and patients only receiving Standard of Care (SOC = red) after 0, 4, 24, 72, and 168 h of study inclusion.

**FIGURE 10:**
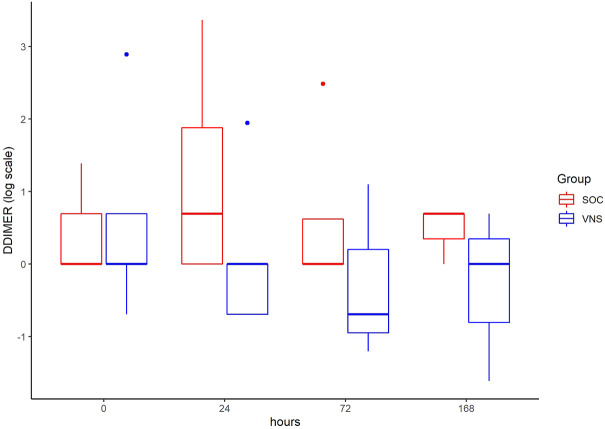
Median DDIMER level with Interquartile Range of patients receiving auricular Vagus Stimulation (VNS = blue) and patients only receiving Standard of Care (SOC = red) after 0, 24, 72, and 168 h of study inclusion.

**FIGURE 11:**
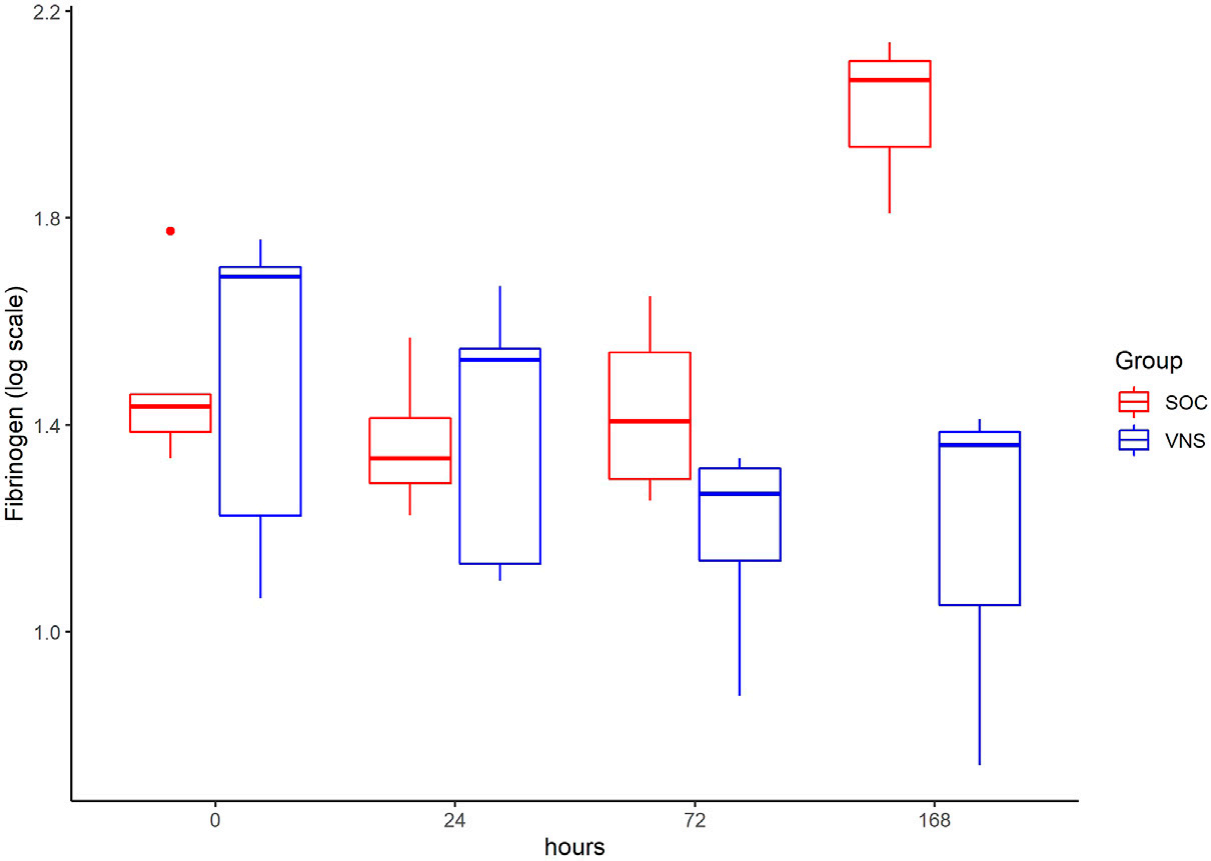
Median Fibrinogen level with Interquartile Range of patients receiving auricular Vagus Stimulation (VNS = blue) and patients only receiving Standard of Care (SOC = red) after 0, 24, 72, and 168 h of study inclusion.

The temporal courses and dynamic ranges of all parameters at each time point and patients are demonstrated in Supplementary Material.

### Difference Inflammation and Clinical Parameters Between VNS and SOC Group

The decrease of IL-6 (*p* = 0.048) and TNFalpha (*p* = 0.048) from T0 to T4 was statistically significantly stronger in VNS than SOC group, as well as the decrease of DDIMER from T0 to T24 (*p* = 0.025). Further, the decrease of CRP from T0 to T72 (*p* = 0.003) was statistically significantly stronger in VNS group, as well as the decrease of CRP (*p* = 0.018) and fibrinogen (*p* = 0.002) from T0 to T168 was statistically significantly stronger in VNS group. On the contrary, the increase of IL-10 from T0 to T72 and T168 was significantly higher in the VNS group (*p* = 0.041 and *p* = 0.048). The exact calculation is shown in Supplementary Material.

**FIGURE 12:**
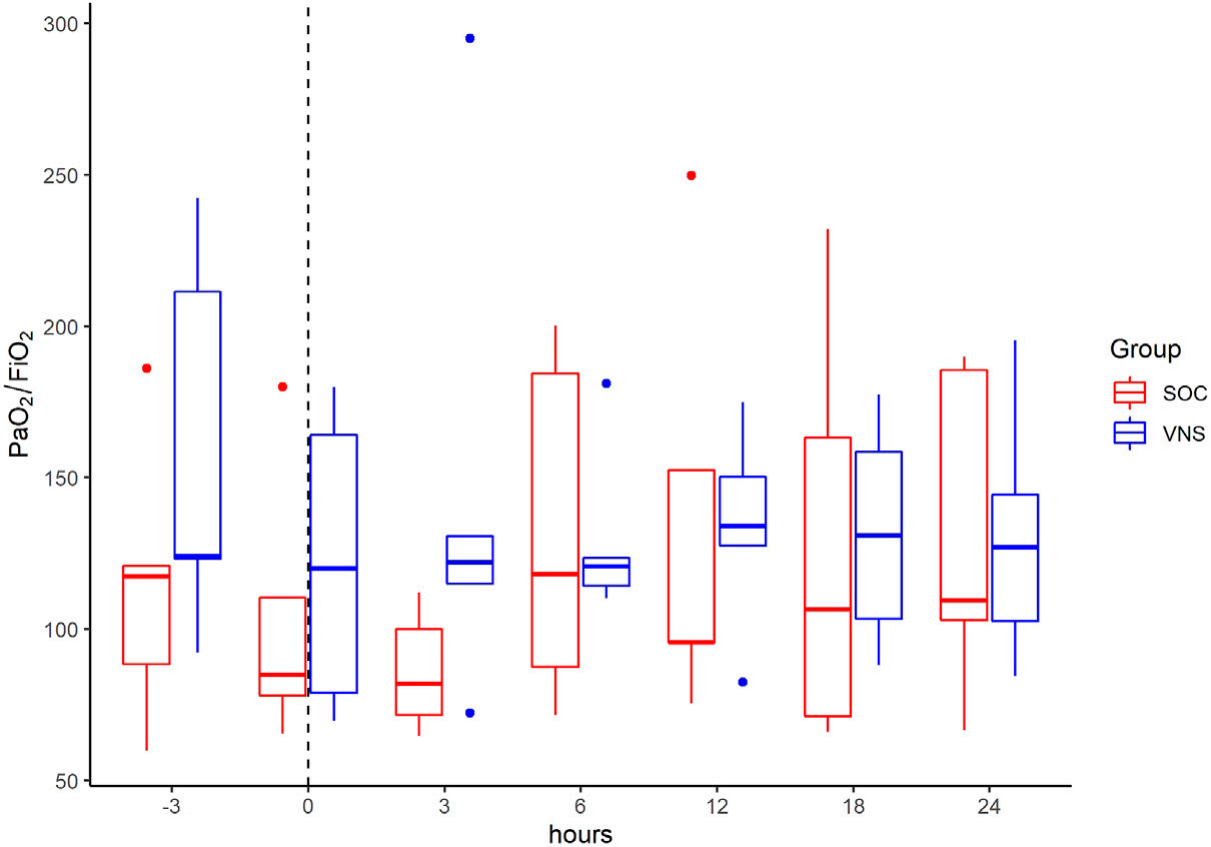
Median Horowitz index (PaO_2_/FiO_2_) with Interquartile Range of patients receiving auricular Vagus Stimulation (VNS = blue) and patients only receiving Standard of Care (SOC = red) prior to, at time of study inclusion (dashed line) and up to 24h afterwards.

An increase of Horowitz index (Pao2/FiO2-Ratio) was seen in the VNS group, as seen in Figure 12.

## Discussion

In this study we evaluated aVNS as a novel procedure to reduce COVID-19 associated cytokine storm. The procedure has been shown to be well tolerated and safe.

Decrease of serum levels of IL-6, TNF Alpha, DDimer between T0 and T4 was significantly more pronounced in the aVNS versus SOC group. Here the mean decrease of TNFα level was 19.3 pg/ml at T0 to 6.4 pg/ml at T4. In contrast, within the control group including patients without aVNS mean TNFα increased from 6.3 pg/ml at T0 to 8.7 pg/ml. The mean decrease of IL-6 level in aVNS group was from 341.4 pg/ml at T0 to 140.2 at T4. In the control group the mean IL-6 rose from 129 pg/ml 131.8 pg/ml. Therefore, aVNS treatment may have a significant impact on patient outcome given that a correlation with worse outcome in Covid-19 has been reported along with high pro-inflammatory levels of IL-6 and TNFα (del Valle et al., 2020). Among others, extensive IL-6 secretion can trigger coagulation and vascular leak syndrome leading to cardiomyopathy, coronary artery disease and myocardial dysfunction (Rabaan et al., 2021).

Conversely, the anti-inflammatory cytokine IL-10 increased in all patients 4 h after the start of aVNS (from 2.7 pg/ml as the mean at T0 to 7 pg/ml at T4) and was significantly higher over time compared to the control group (from 8.6 pg/ml to 8.6 pg/ml). However, in all patients the levels were only slightly elevated. Extremely high and low levels of IL-10 are considered to play a role in the cytokine storm of COVID-19 (Rabaan et al., 2021). The clinical impact of the slight elevation of IL-10 after receiving aVNS is not clear so far.

In all patients the nonspecific inflammation biomarker CRP decreased (from a mean level of 151.9 mg/dl at T0 to 31.5 mg/dl at T7) while receiving aVNS. In comparison to patients who were not treated with aVNS the mean CRP levels increased (from 83.9 mg/dl to 187.1 mg/dl). Ferritin decreased as well initially, but there was no clear difference in comparison to the control group. In all patients, the inflammation and coagulation biomarkers DDIMER (from 4.5 μg/ml at T0 to 1.5 μg/ml at T168) and fibrinogen (from 4.6 g/L at T0 to 3.7 g/lat T168) decreased initially after the start of aVNS, while in the control group the mean fibrinogen levels increased from 4.4 g/L to 7.5 g/L and mean DDIMER levels decreased from significantly less from 1.8 μg/ml to 1.7 μg/ml. It is not known that the patients receiving aVNS have a better clinical outcome due to the lower inflammation parameters; however, in another study elevated CRP, ferritin, and DDIMER were found to predict worsening outcomes in Covid-19 (Caricchio et al., 2021), which may indicate a favourable effect of aVNS. Other studies have shown that a decrease of inflammation parameters throughout the clinical course improved patient outcomes in Covid-19 (Zhou et al., 2020) and reduced risk and severity of ARDS (Wu et al., 2020). Furthermore, in this study a decrease of macrophage activation parameters such as sIL-R, calprotectin, and IL-18 in all patients 4 h after initiation of aVNS was observed, in contrast to the SOC group, in which an increase by 40–60% was registered.

It is noteworthy that the early aVNS-induced decline of proinflammatory mediators such as IL-6 was not sustained during the observation period. aVNS most likely has a complex effect on the regulation of the inflammatory cascade during COVID-19. Along these lines it is interesting to note that aVNS did not have a down-regulatory effect on all proinflammatory parameters alike. While IL-6 and TNF-alpha decreased early following aVNS treatment, other mediators of phagocyte activation such as calprotectin or IL-18 seemed to be less affected. Calprotectin has crucial activities in the regulation of immune homeostasis and inflammation and triggers inflammation through interaction—as an endogenous agonist - with the proinflammatory phagocyte receptor TLR4. Binding to TLR4 initiates a signaling cascade including IL-6 expression in an NF-κB-dependent manner (Vogl et al., 2007). Through induction of IFN-gamma IL-18 primes mononuclear phagocytes for TNF-alpha-induced IL-6 expression, which might be particularly relevant during the later stages of viral infection. Independently of IFNγ or other cytokines, IL-18 exhibits proinflammatory characteristics such as increases in cell adhesion molecules, nitric oxide synthesis, and chemokine production via NF-kB activation (Kohka et al., 1998). It is thus possible that aVNS has a direct effect on the early phase of IL-6 induction while the effect on a later phase of IL-6 release that is predominantly secondary to other proinflammatory mediators is less pronounced. Alternatively, desensitization of the aVNS effect on IL-6 production might develop, as has been described for vagus nerve stimulation-induced weight loss (Khan et al., 2017), probably involving desensitisation of adrenergic receptors involved in the therapeutic effect, a process that leads to reduced receptor responsiveness after prolonged stimulation (Carrara et al., 2021).

To the best of our knowledge, this is the first prospective randomised study using aVNS in patients with critical Covid-19. Although the potential positive impact of vagus nerve stimulation on cytokine storm in patients with severe Covid-19 was often discussed (Bonaz et al., 2020a, 2020b; Bara, de Ridder and Maciaczyk, 2020; Kaniusas and Szeles, 2020b; Guo et al., 2021; Wang et al., 2022), clinical data is scarce. Boezaart et al. reported two cases of patients with severe Covid-19 receiving transcutaneous aVNS in addition to standard of care in which case a rapid decrease of IL-6 was observed and aVNS was well tolerated (Boezaart and Botha, 2021). However, in contrast to our study, there was no comparison to a control group. Further, in our study percutaneous stimulation with miniature needle electrodes was preferred over the transcutaneous stimulation with surface electrodes in order to avoid diffuse stimulation of vagal and non-vagal nerves in the auricle as well as to avoid large stimulation voltages (to overcome skin barrier). In addition, an uncomfortable tragus clip was also avoided for the comfort of the patient.

At this point in time the Covid-19 pandemic has been ongoing for more than 2 years and the clinical presentation of the disease is known to vary widely. Although most cases of Covid-19 are mild or asymptomatic, in some cases the initial stage of viral replication can be followed by a stage of hyperinflammatory response to SARS-CoV-2 infection resulting in severe disease with acute respiratory distress syndrome (ARDS) or even multi-organ failure (Fajgenbaum and June 2020). The pathomechanism of the ‘cytokine storm’ is not fully explained. Tay et al. (Tay et al., 2020) postulated that SARS-CoV-2 is a cytopathic virus inducing death of infected cells during viral replication due to pyroptosis: an inflammatory form of programmed cell death caused by secretion of proinflammatory cytokines such as IL-6 and IFNγ. The excessive secretion of cytokines recruits activated macrophages and T cells to the site of infection inducing local inflammation that could lead to tissue damage. This local inflammation again stimulates systemic cytokine production further escalating the cytokine storm. This excessive inflammatory response can cause further local tissue damage, like destruction of lung parenchyma resulting in ARDS and multi-organ failure. Furthermore, the excessive recruitment of activated macrophages can lead to hemophagocytosis which can promote organ failure at several sites. Postmortem analysis has demonstrated the presence of hemophagocytosis in lung, heart, liver, bone marrow, and the reticuloendothelial organs (Fox et al., 2020; Bryce et al., 2021) in patients with severe Covid-19.

Several medications have been developed to reduce cytokine expression or systemic inflammation, such as antibodies against different interleukin receptors or cortisone. However, interference with the immune system can cause an array of different problems such as increased risk of secondary infection (Fajgenbaum and June 2020).

The non-invasive vagus nerve stimulation on the neck has shown a decrease in inflammatory markers such as CRP and procalcitonin, as stated in a non peer-reviewed preprint (Tornero et al., 2021a; 2021b), whereas this type of stimulation was successfully applied in managing respiratory symptoms in two case studies (Staats et al., 2020; Tornero et al., 2021a). In general, neuromodulatory applications have a strong rationale for their use in acute and chronic Covid-19 symptoms (Pilloni et al., 2020) along various pathways, such as modulation of anti-inflammatory responses, amelioration of musculoskeletal pain and fatigue, augmenting rehabilitation, and reducing mental distress (Baptista et al., 2020). Antinociceptive effects of aVNS were also demonstrated, in line with the use of the same stimulation device in chronic cervical pain and chronic low-back pain (Sator-Katzenschlager et al., 2003, 2004).

Limitations of the study are the lack of interpretation of clinical outcome and the small number of participants. Further we did not include many clinical parameters. We showed an increase of Horowitz Index after start of VNS. However, interpretation must be cautious, because it can be influenced by many factors, like breathing index or ventilation method. These limitations should be addressed in future studies.

In summary, this study shows that aVNS has the potential to reduce expression of pro-inflammatory proteins and increase expression of anti-inflammatory proteins in patients with severe Covid-19. Given the good tolerance and low risk of side effects, non-invasive aurical vagus stimulation might present a good option for additional treatment of patients with hyperinflammatory Covid-19.

## Data Availability

The original contributions presented in the study are included in the article/Supplementary Material, further inquiries can be directed to the corresponding author.
