# Supplementary material for: Percutaneous Auricular Vagus Nerve Stimulation Reduces Inflammation in Critical Covid-19 Patients
Source: Front Physiol. 2022 Jul 4;13:897257. doi: 10.3389/fphys.2022.897257 (PMC9289290; doi:10.3389/fphys.2022.897257)
Supplement: Supplementary file 1 [file DataSheet1.docx]

Supplementary Material

## 1 Supplementary Figures


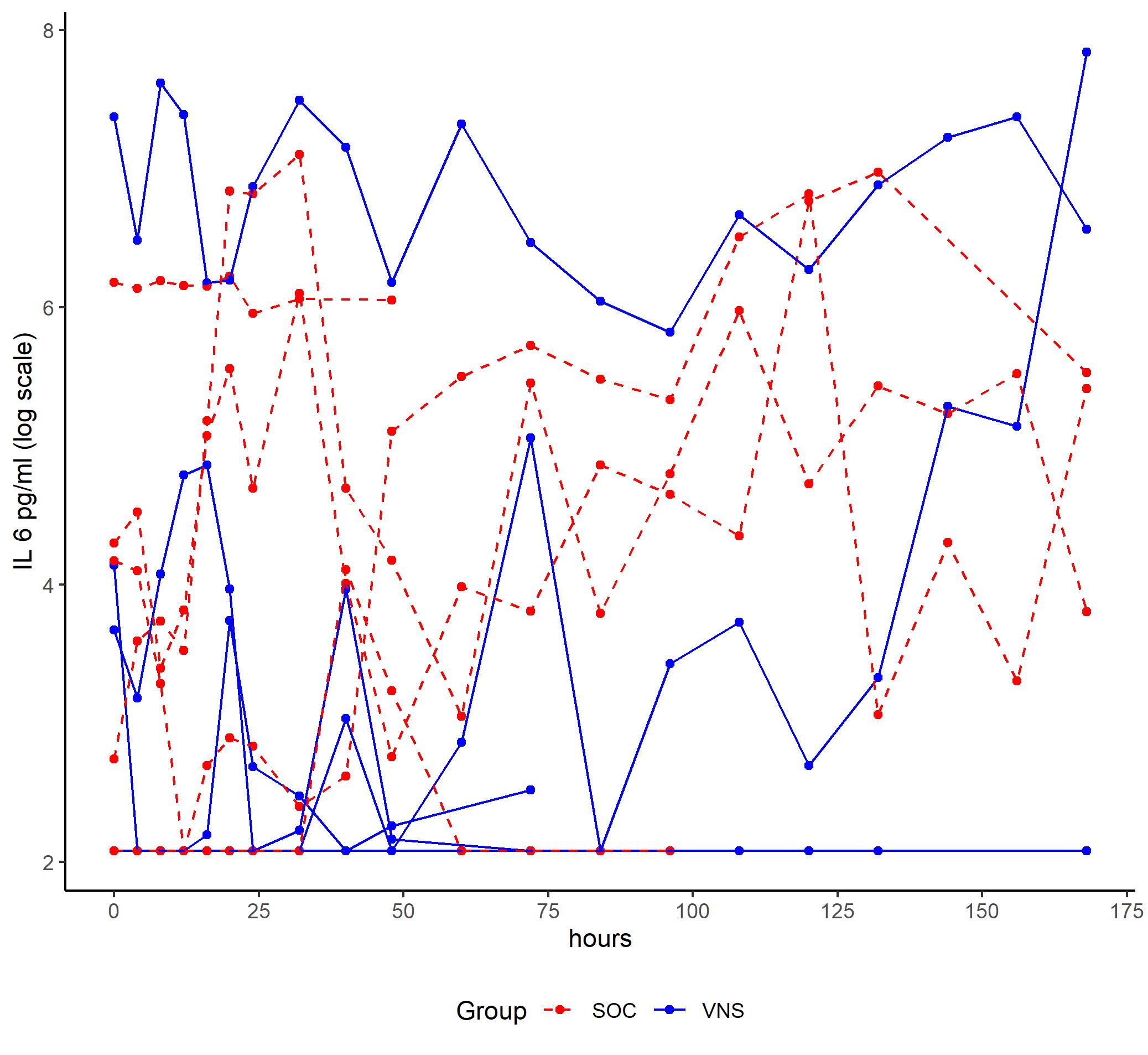


**Supplementary Figure 1.** Dynamic of IL-6 level in serum in 5 patients receiving auricular Vagus Stimulation (VNS = blue) and 5 patients only receiving Standard of Care (SOC = red).


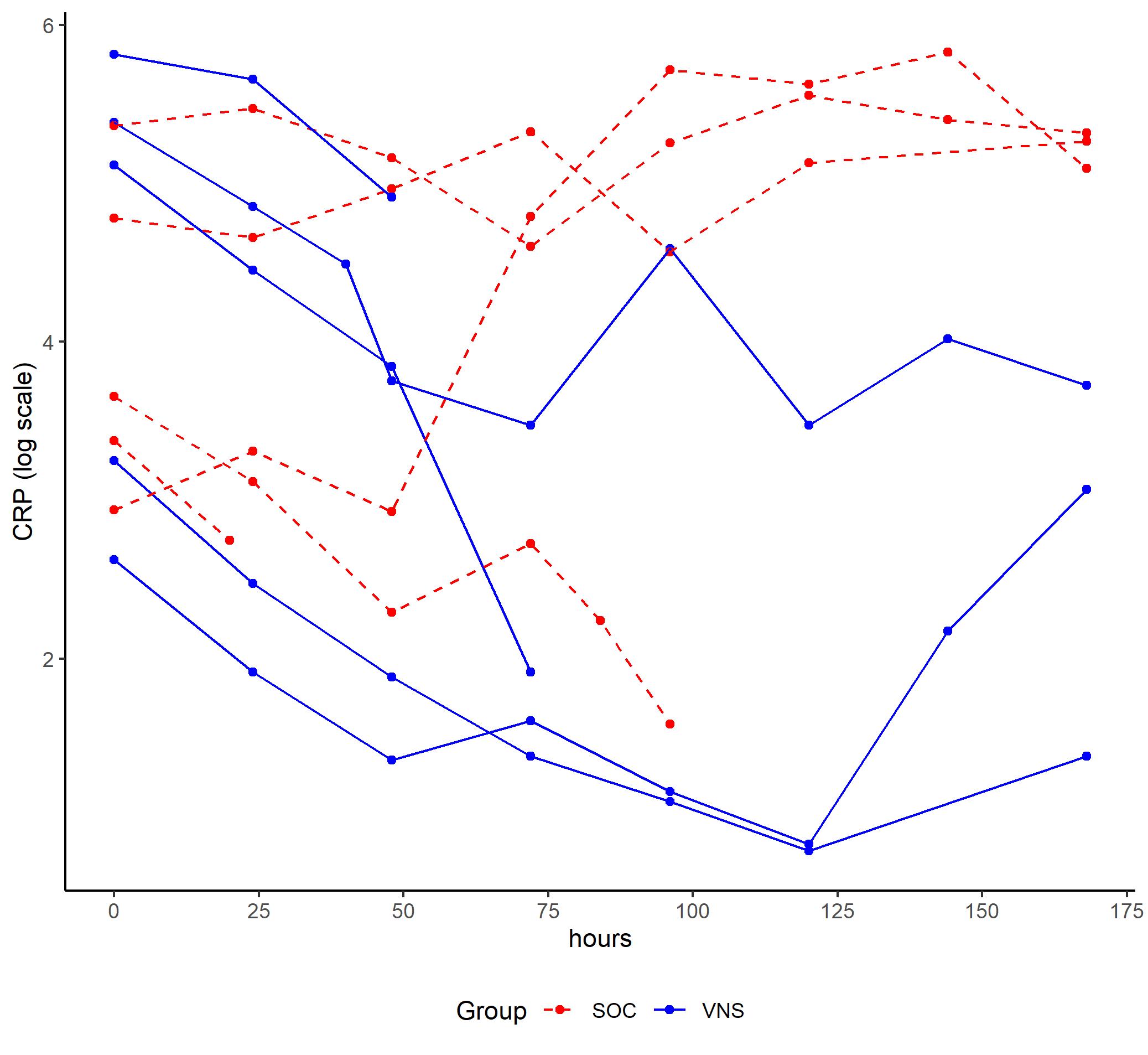


**Supplementary Figure 2.** Dynamic of CRP level in serum in 5 patients receiving auricular Vagus Stimulation (VNS = blue) and 5 patients only receiving Standard of Care (SOC = red).


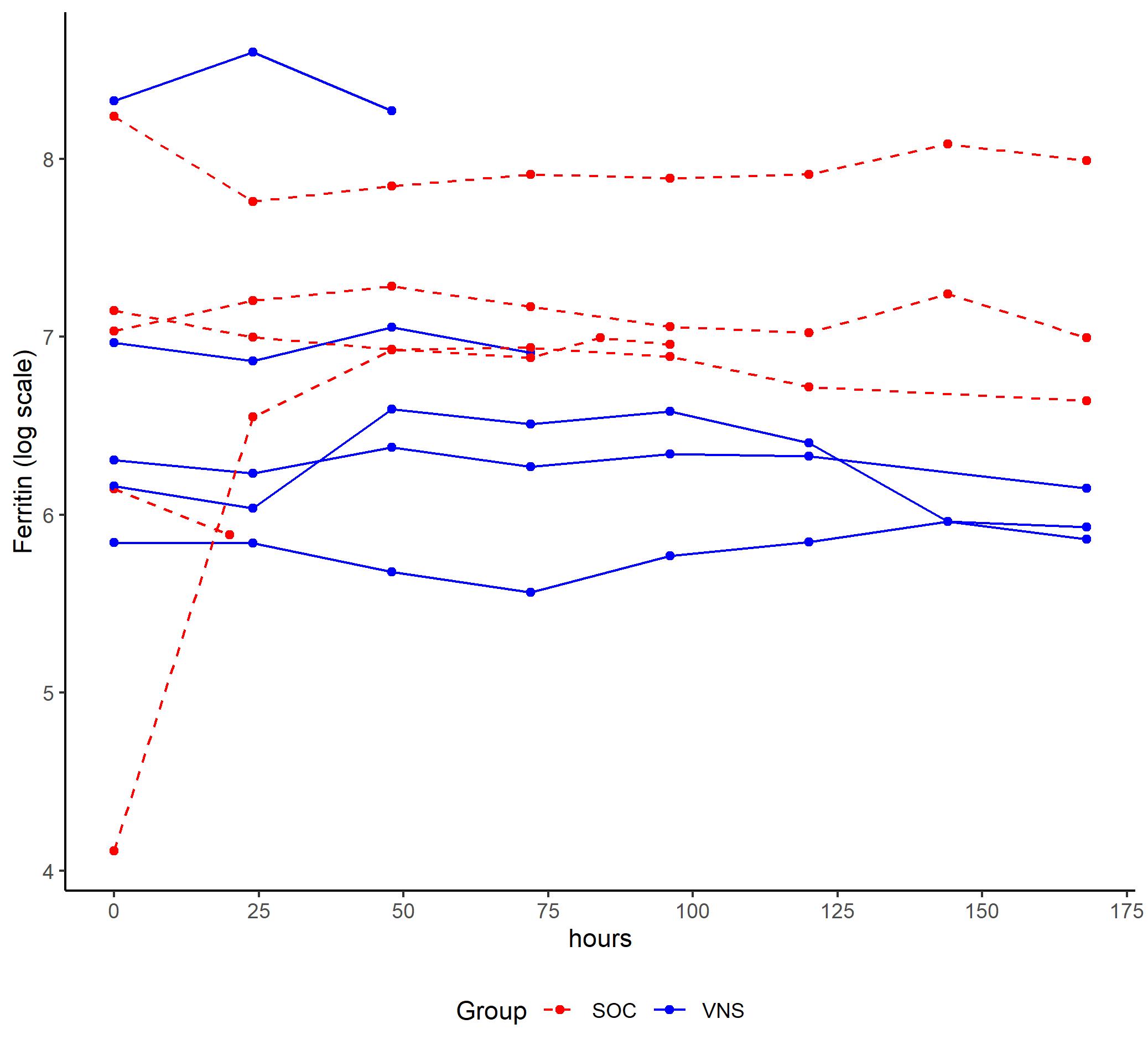


**Supplementary Figure 3.** Dynamic of ferritin level in serum in 5 patients receiving auricular Vagus Stimulation (VNS = blue) and 5 patients only receiving Standard of Care (SOC = red).


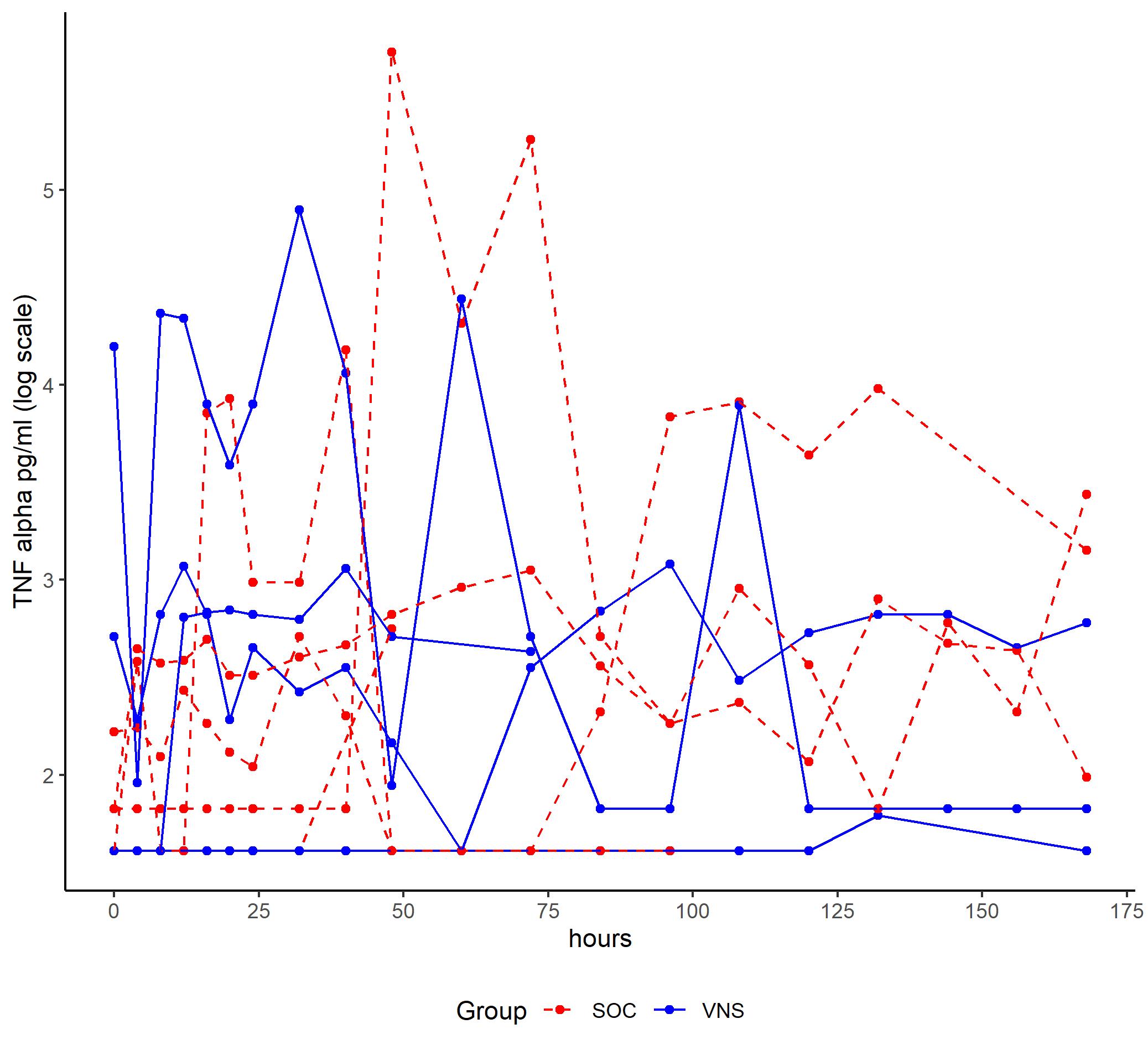


**Supplementary Figure 4.** Dynamic of TNFalpha level in serum in 5 patients receiving auricular Vagus Stimulation (VNS = blue) and 5 patients only receiving Standard of Care (SOC = red).


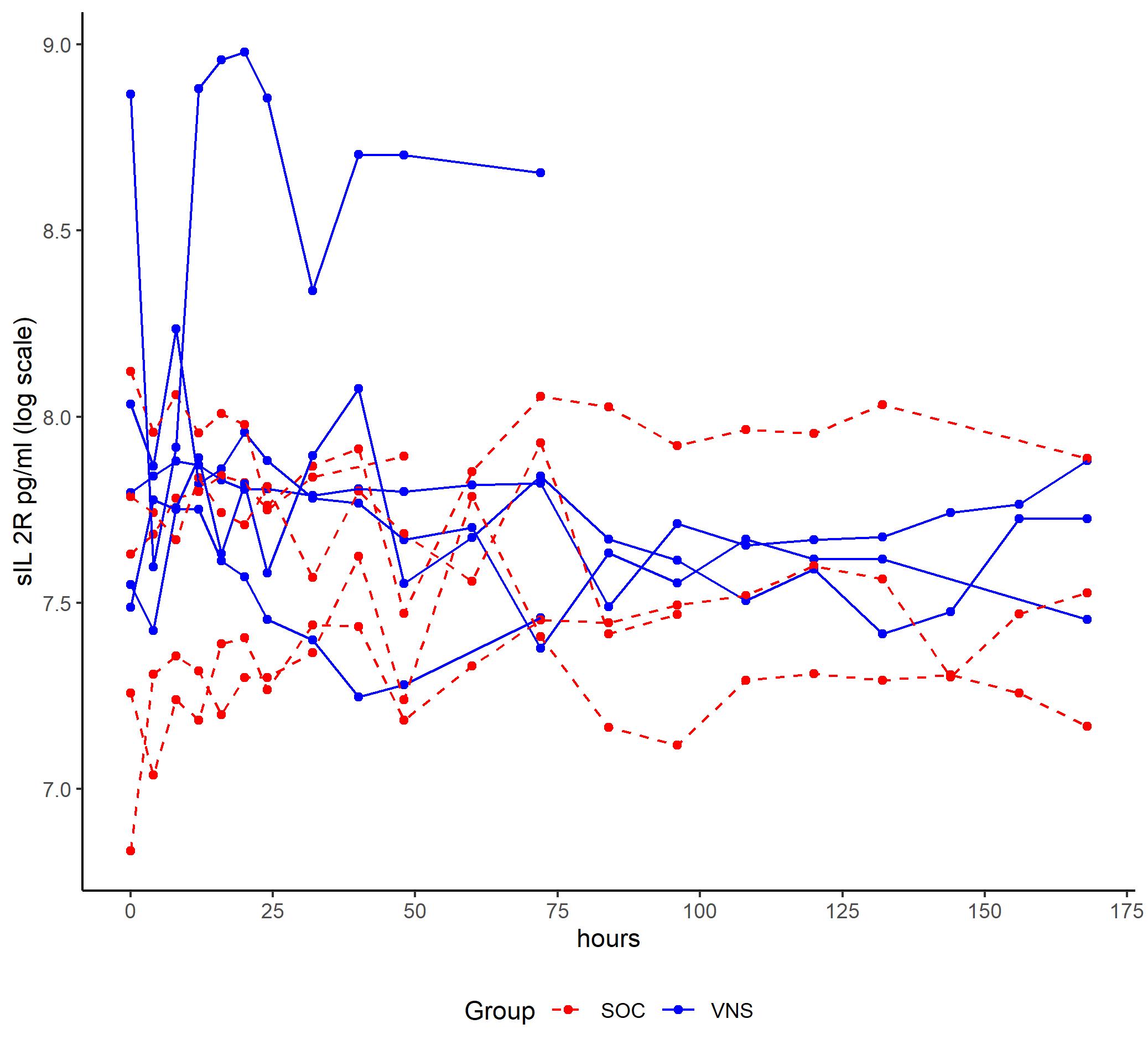


**Supplementary Figure 5.** Dynamic of sIL2R level in serum in 5 patients receiving auricular Vagus Stimulation (VNS = blue) and 5 patients only receiving Standard of Care (SOC = red).


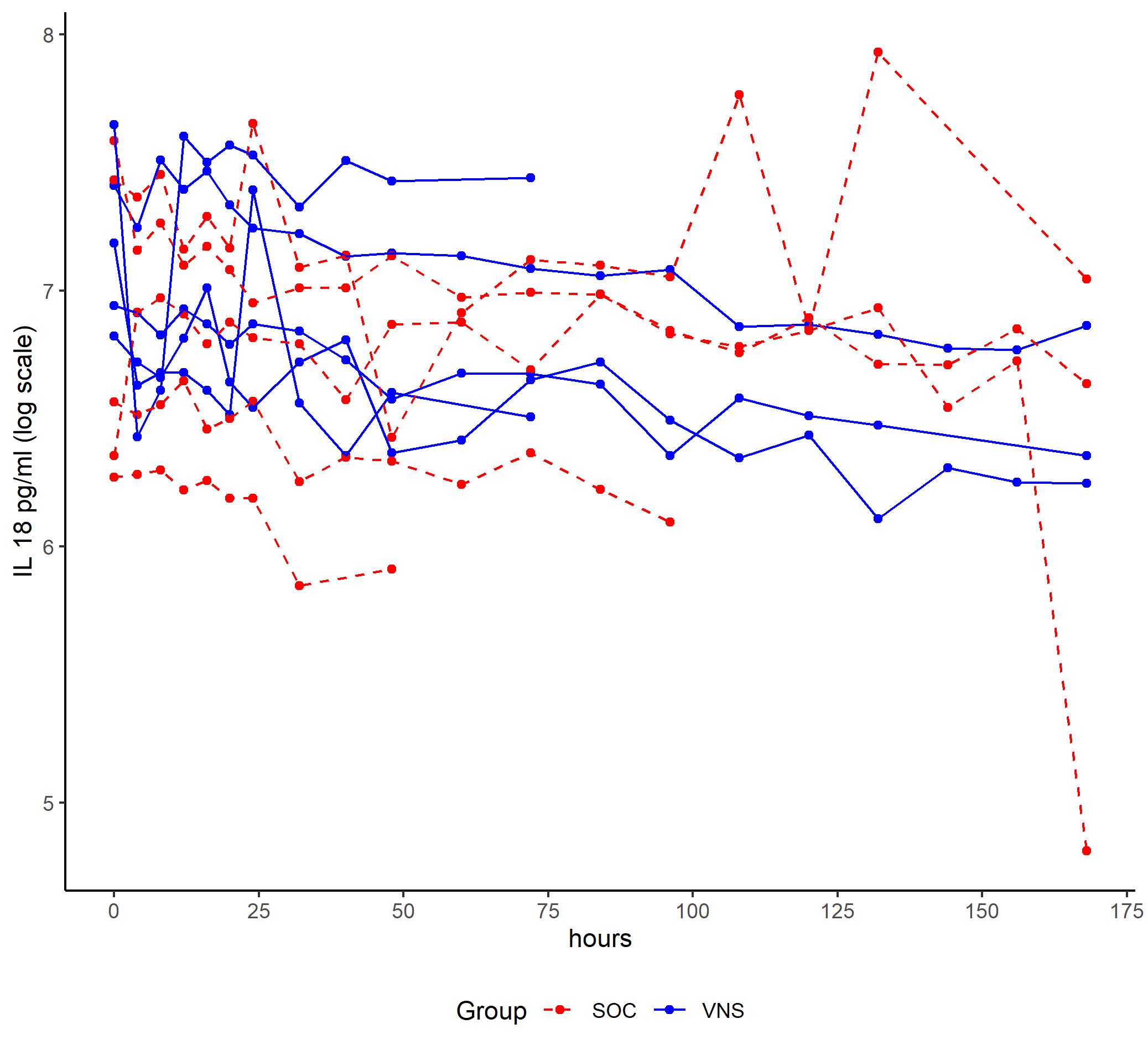


**Supplementary Figure 6.** Dynamic of IL-18 level in serum in 5 patients receiving auricular Vagus Stimulation (VNS = blue) and 5 patients only receiving Standard of Care (SOC = red).


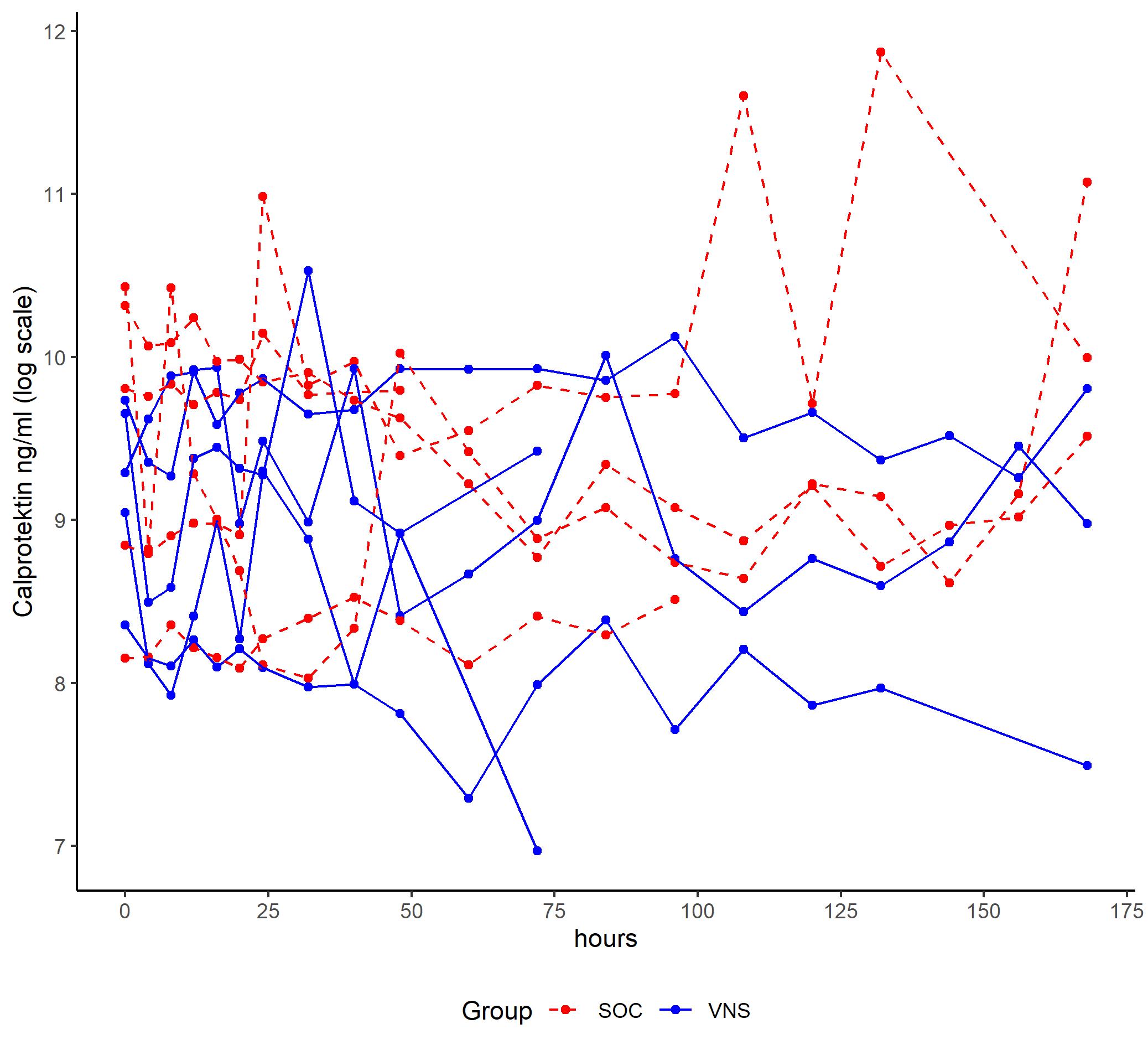


**Supplementary Figure 7.** Dynamic of Calprotektin level in serum in 5 patients receiving auricular Vagus Stimulation (VNS = blue) and 5 patients only receiving Standard of Care (SOC = red).


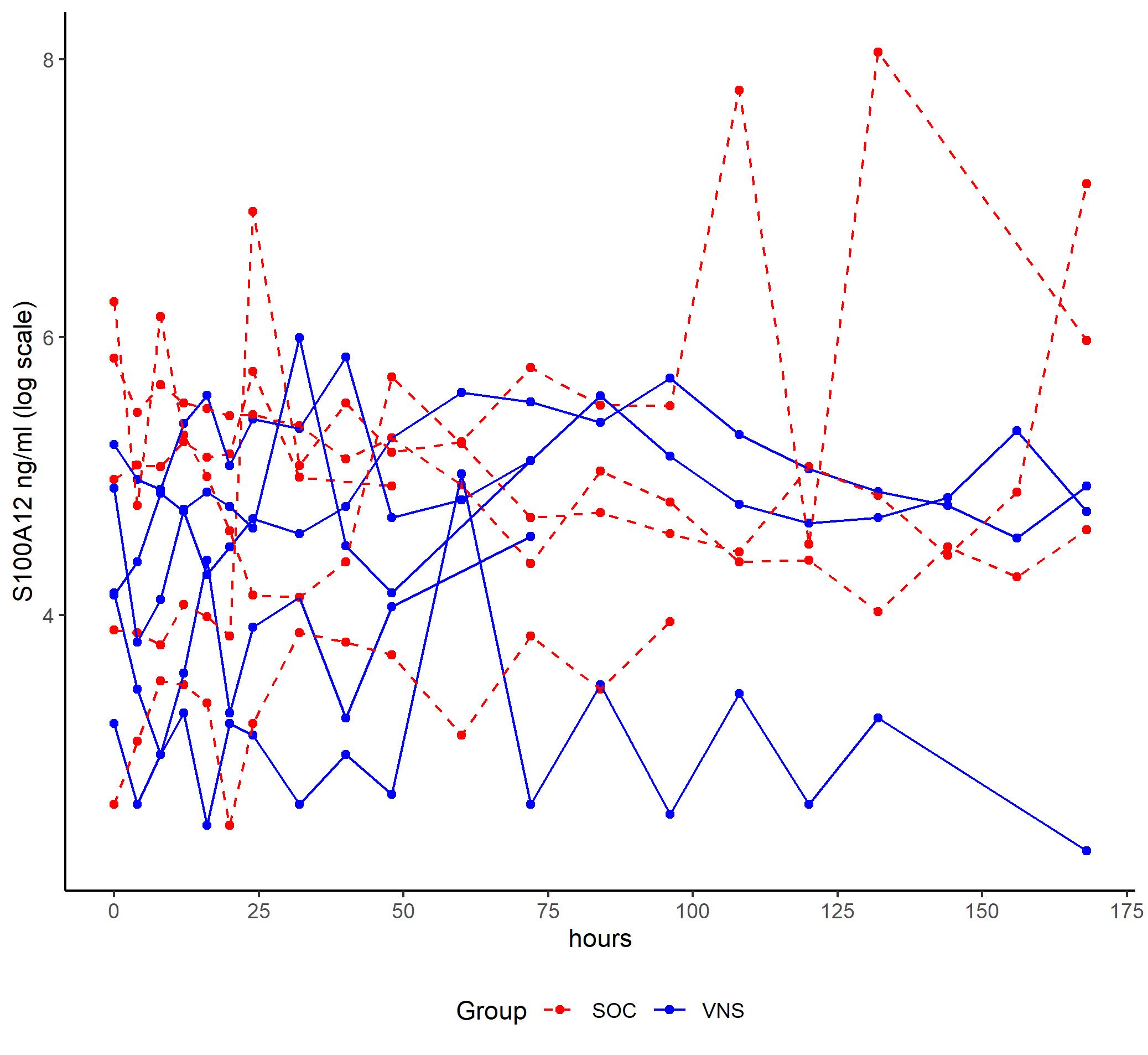


**Supplementary Figure 8.** Dynamic of S100A12 level in serum in 5 patients receiving auricular Vagus Stimulation (VNS = blue) and 5 patients only receiving Standard of Care (SOC = red).


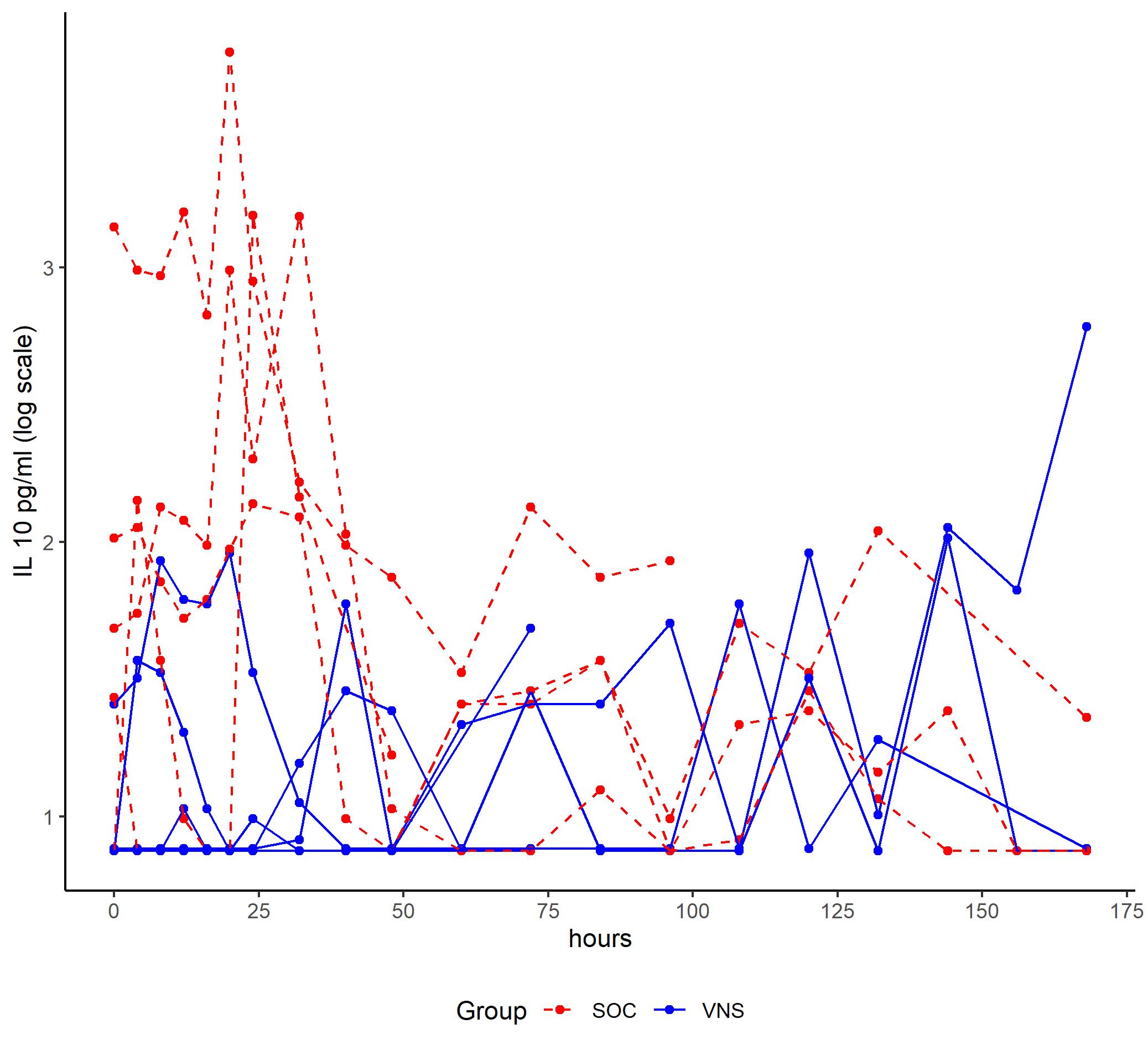


**Supplementary Figure 9.** Dynamic of IL-10 level in serum in 5 patients receiving auricular Vagus Stimulation (VNS = blue) and 5 patients only receiving Standard of Care (SOC = red).


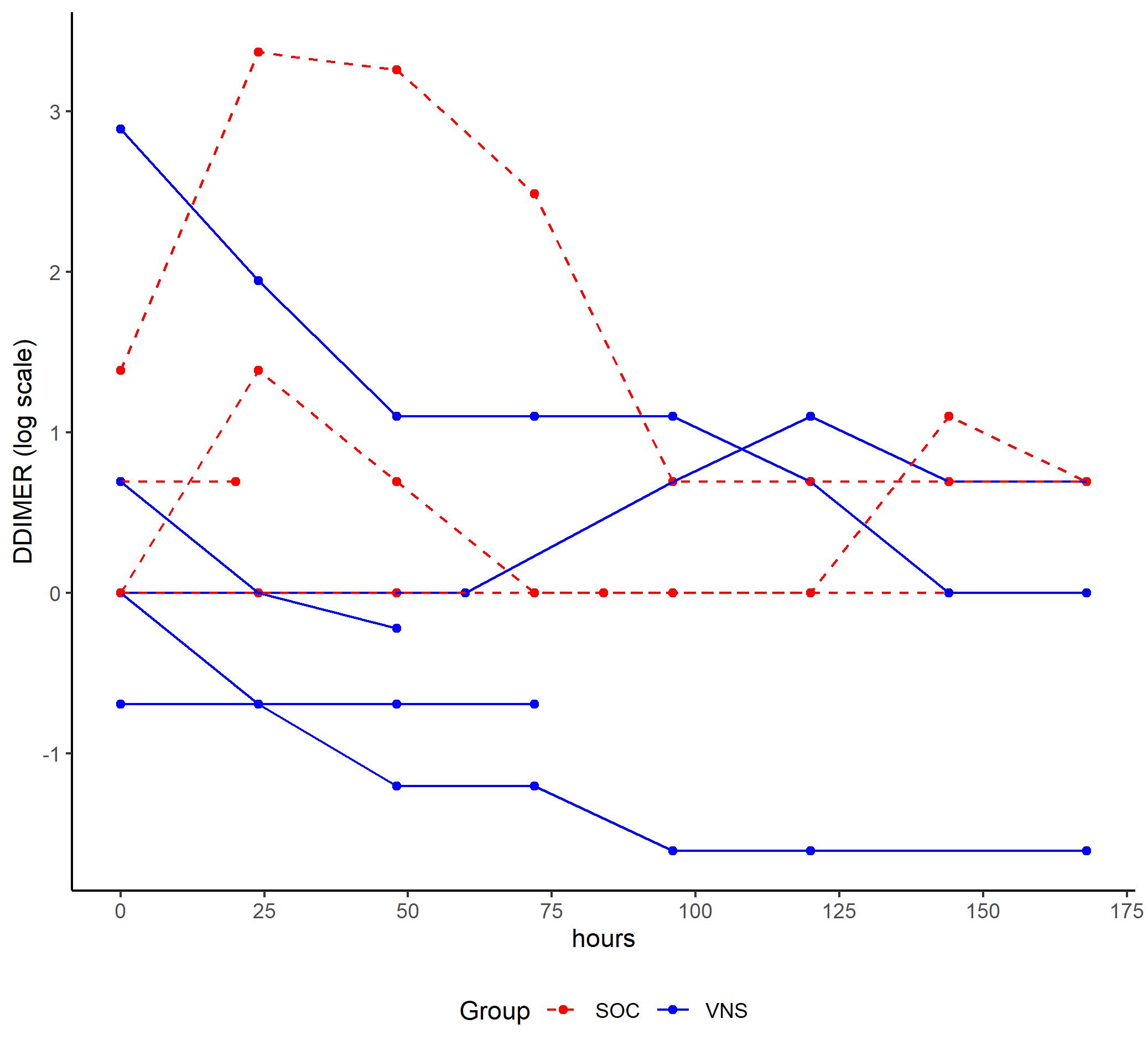


**Supplementary Figure 10**Dynamic of DDIMER level in serum in 5 patients receiving auricular Vagus Stimulation (VNS = blue) and 5 patients only receiving Standard of Care (SOC = red).


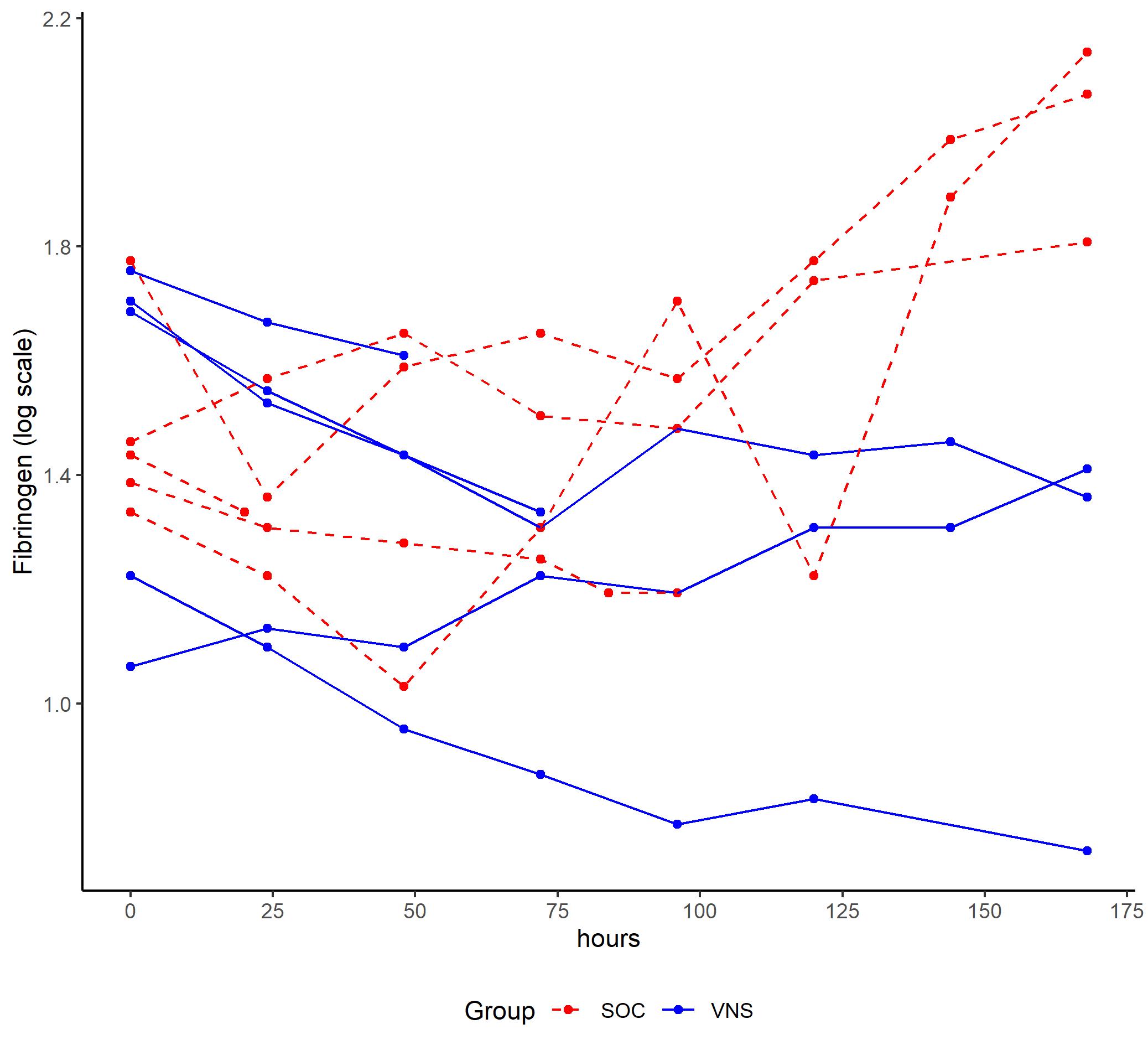


**Supplementary Figure 11.** Dynamic of fibrinogen level in serum in 5 patients receiving auricular Vagus Stimulation (VNS = blue) and 5 patients only receiving Standard of Care (SOC = red).

# Supplementary Tables

| **Parameter** | **Value** | **Std.Error** | **DF** | **t-value** | **p-value** |
| --- | --- | --- | --- | --- | --- |
| *(Intercept)* | 1.817 | 0.367 | 27 | 4.951 | <0.001 |
| *T4 vs. T0* | 0.363 | 0.340 | 27 | 1.066 | 0.296 |
| *T24 vs. T0* | 0.377 | 0.458 | 27 | 0.823 | 0.418 |
| *T72 vs. T0* | 0.966 | 0.718 | 27 | 1.345 | 0.190 |
| *T72 vs. T0* | 0.910 | 0.529 | 27 | 1.719 | 0.097 |
| *Group VNS vs. SOC* | 0.529 | 0.519 | 8 | 1.019 | 0.338 |
| *T4 vs. T0 x Group* | -0.895 | 0.481 | 27 | -1.860 | 0.005* |
| *T24 vs. T0 x Group* | -0.204 | 0.648 | 27 | -0.316 | 0.755 |
| *T72 vs. T0 x Group* | -1.091 | 0.978 | 27 | -1.115 | 0.275 |
| *T72 vs. T0 x Group* | -1.239 | 0.747 | 27 | -1.658 | 0.109 |

**Supplementary Table 1.** TNF-alpha (log-scale) after 4, 24, 72 and 168 compared to the baseline T0 of patients in VNS in comparison to SOC. *difference statistically significant

| **Parameter** | **Value** | **Std.Error** | **DF** | **t-value** | **p-value** |
| --- | --- | --- | --- | --- | --- |
| *(Intercept)* | 3.895 | 0.815 | 27 | 4.777 | <0.001 |
| *T4 vs. T0* | 0.191 | 0.297 | 27 | 0.645 | 0.525 |
| *T24 vs. T0* | 0.581 | 0.661 | 27 | 0.879 | 0.387 |
| *T72 vs. T0* | 0.847 | 0.585 | 27 | 1.448 | 0.159 |
| *T72 vs. T0* | 0.899 | 1.057 | 27 | 0.850 | 0.403 |
| *Group VNS vs. SOC* | -0.027 | 1.153 | 8 | -0.023 | 0.982 |
| *T4 vs. T0 x Group* | **-0.878** | **0.419** | **27** | **-2.094** | **0.046*** |
| *T24 vs. T0 x Group* | -1.289 | 0.935 | 27 | -1.379 | 0.179 |
| *T72 vs. T0 x Group* | -1.075 | 0.797 | 27 | -1.348 | 0.189 |
| *T72 vs. T0 x Group* | -0.085 | 1.490 | 27 | -0.057 | 0.955 |

**Supplementary Table 2.** IL-6 (log-scale) after 4, 24, 72 and 168 compared to the baseline T0 of patients in VNS in comparison to SOC. *difference statistically significant

| **Parameter** | **Value** | **Std.Error** | **DF** | **t-value** | **p-value** |
| --- | --- | --- | --- | --- | --- |
| *(Intercept)* | 4.023 | 0.536 | 17 | 7.511 | <0.001 |
| *T24 vs. T0* | -0.018 | 0.343 | 17 | -0.053 | 0.958 |
| *T72 vs. T0* | 0.227 | 0.461 | 17 | 0.492 | 0.629 |
| *T72 vs. T0* | 0.657 | 0.571 | 17 | 1.150 | 0.266 |
| *Group VNS vs. SOC* | 0.415 | 0.758 | 8 | 0.548 | 0.599 |
| *T24 vs. T0 x Group* | -0.548 | 0.462 | 17 | -1.187 | 0.252 |
| *T72 vs. T0 x Group* | **-2.196** | **0.640** | **17** | **-3.429** | **0.003*** |
| *T72 vs. T0 x Group* | **-2.108** | **0.801** | **17** | **-2.631** | **0.018*** |

**Supplementary Table 3.** CRP (log-scale) after 24, 72 and 168 compared to the baseline T0 of patients in VNS in comparison to SOC. *difference statistically significant

| **Parameter** | **Value** | **Std.Error** | **DF** | **t-value** | **p-value** |
| --- | --- | --- | --- | --- | --- |
| *(Intercept)* | 1.832 | 0.241 | 27 | 7.588 | <0.001 |
| *T4 vs. T0* | 0.130 | 0.258 | 27 | 0.506 | 0.617 |
| *T24 vs. T0* | 0.460 | 0.320 | 27 | 1.438 | 0.162 |
| *T72 vs. T0* | -0.434 | 0.244 | 27 | -1.783 | 0.086 |
| *T72 vs. T0* | -0.631 | 0.393 | 27 | -1.605 | 0.120 |
| *Group VNS vs. SOC* | -0.848 | 0.341 | 8 | -2.483 | 0.038 |
| *T4 vs. T0 x Group* | 0.027 | 0.365 | 27 | 0.073 | 0.942 |
| *T24 vs. T0 x Group* | -0.413 | 0.452 | 27 | -0.914 | 0.369 |
| *T72 vs. T0 x Group* | **0.713** | **0.332** | **27** | **2.148** | **0.041*** |
| *T72 vs. T0 x Group* | **1.150** | **0.556** | **27** | **2.069** | **0.048*** |

**Supplementary Table 4.** IL-10 (log-scale) after 4, 24, 72 and 168 compared to the baseline T0 of patients in VNS in comparison to SOC. *difference statistically significant

| **Parameter** | **Value** | **Std.Error** | **DF** | **t-value** | **p-value** |
| --- | --- | --- | --- | --- | --- |
| *(Intercept)* | 7.525 | 0.188 | 27 | 40.104 | <0.001 |
| *T4 vs. T0* | 0.020 | 0.161 | 27 | 0.126 | 0.901 |
| *T24 vs. T0* | 0.052 | 0.161 | 27 | 0.325 | 0.748 |
| *T72 vs. T0* | 0.215 | 0.174 | 27 | 1.239 | 0.226 |
| *T72 vs. T0* | 0.105 | 0.192 | 27 | 0.546 | 0.590 |
| *Group VNS vs. SOC* | 0.421 | 0.265 | 8 | 1.587 | 0.151 |
| *T4 vs. T0 x Group* | -0.266 | 0.228 | 27 | -1.166 | 0.254 |
| *T24 vs. T0 x Group* | -0.083 | 0.228 | 27 | -0.364 | 0.719 |
| *T72 vs. T0 x Group* | -0.330 | 0.237 | 27 | -1.395 | 0.174 |
| *T72 vs. T0 x Group* | -0.264 | 0.271 | 27 | -0.975 | 0.338 |

**Supplementary Table 5.** sIL-2R (log-scale) after 4, 24, 72 and 168 compared to the baseline T0 of patients in VNS in comparison to SOC. *difference statistically significant

| **Parameter** | **Value** | **Std.Error** | **DF** | **t-value** | **p-value** |
| --- | --- | --- | --- | --- | --- |
| *(Intercept)* | 6.842 | 0.201 | 27 | 34.007 | <0.001 |
| *T4 vs. T0* | 0.005 | 0.184 | 27 | 0.027 | 0.979 |
| *T24 vs. T0* | -0.006 | 0.160 | 27 | -0.039 | 0.969 |
| *T72 vs. T0* | -0.177 | 0.147 | 27 | -1.203 | 0.239 |
| *T72 vs. T0* | -0.936 | 0.519 | 27 | -1.804 | 0.082 |
| *Group VNS vs. SOC* | 0.360 | 0.285 | 8 | 1.266 | 0.241 |
| *T4 vs. T0 x Group* | -0.419 | 0.260 | 27 | -1.615 | 0.118 |
| *T24 vs. T0 x Group* | -0.080 | 0.226 | 27 | -0.354 | 0.726 |
| *T72 vs. T0 x Group* | -0.153 | 0.201 | 27 | -0.760 | 0.454 |
| *T72 vs. T0 x Group* | 0.302 | 0.733 | 27 | 0.411 | 0.684 |

**Supplementary Table 6.** IL-18 (log-scale) after 4, 24, 72 and 168 compared to the baseline T0 of patients in VNS in comparison to SOC. *difference statistically significant

| **Parameter** | **Value** | **Std.Error** | **DF** | **t-value** | **p-value** |
| --- | --- | --- | --- | --- | --- |
| *(Intercept)* | 9.510 | 0.405 | 27 | 23.500 | <0.001 |
| *T4 vs. T0* | -0.389 | 0.371 | 27 | -1.051 | 0.303 |
| *T24 vs. T0* | -0.039 | 0.451 | 27 | -0.086 | 0.933 |
| *T72 vs. T0* | -0.436 | 0.511 | 27 | -0.853 | 0.401 |
| *T72 vs. T0* | 0.609 | 0.574 | 27 | 1.062 | 0.298 |
| *Group VNS vs. SOC* | -0.295 | 0.572 | 8 | -0.515 | 0.621 |
| *T4 vs. T0 x Group* | -0.078 | 0.524 | 27 | -0.149 | 0.883 |
| *T24 vs. T0 x Group* | 0.027 | 0.637 | 27 | 0.043 | 0.966 |
| *T72 vs. T0 x Group* | -0.119 | 0.704 | 27 | -0.168 | 0.868 |
| *T72 vs. T0 x Group* | -1.234 | 0.806 | 27 | -1.530 | 0.138 |

**Supplementary Table 7.** Calprotektin (log-scale) after 4, 24, 72 and 168 compared to the baseline T0 of patients in VNS in comparison to SOC. *difference statistically significant

| **Parameter** | **Value** | **Std.Error** | **DF** | **t-value** | **p-value** |
| --- | --- | --- | --- | --- | --- |
| *(Intercept)* | 4.722 | 0.503 | 27 | 9.382 | <0.001 |
| *4 hours vs baseline* | -0.264 | 0.374 | 27 | -0.706 | 0.486 |
| *24 hours vs baseline* | 0.370 | 0.482 | 27 | 0.768 | 0.449 |
| *72 hours vs baseline* | 0.072 | 0.569 | 27 | 0.127 | 0.900 |
| *168 hours vs baseline* | 1.040 | 0.655 | 27 | 1.588 | 0.124 |
| *VNS vs SOC* | -0.390 | 0.712 | 8 | -0.548 | 0.598 |
| *4 hours vs baseline x Group* | -0.214 | 0.529 | 27 | -0.404 | 0.689 |
| *24 hours vs baseline x Group* | -0.347 | 0.682 | 27 | -0.509 | 0.615 |
| *72 hours vs baseline x Group* | 0.188 | 0.786 | 27 | 0.239 | 0.813 |
| *168 hours vs baseline x Group* | -1.264 | 0.919 | 27 | -1.376 | 0.180 |

**Supplementary Table 8.** S100A12 (log-scale) after 4, 24, 72 and 168 compared to the baseline T0 of patients in VNS in comparison to SOC. *difference statistically significant

| **Parameter** | **Value** | **Std.Error** | **DF** | **t-value** | **p-value** |
| --- | --- | --- | --- | --- | --- |
| *(Intercept)* | 0.416 | 0.472 | 16 | 0.881 | 0.391 |
| *T24 vs. T0* | 0.823 | 0.385 | 16 | 2.140 | 0.048 |
| *T72 vs. T0* | 0.242 | 0.500 | 16 | 0.484 | 0.635 |
| *T72 vs. T0* | -0.078 | 0.605 | 16 | -0.129 | 0.899 |
| *Group VNS vs. SOC* | 0.162 | 0.668 | 8 | 0.243 | 0.814 |
| *T24 vs. T0 x Group* | **-1.289** | **0.519** | **16** | **-2.483** | **0.025*** |
| *T72 vs. T0 x Group* | -0.948 | 0.719 | 16 | -1.319 | 0.206 |
| *T72 vs. T0 x Group* | -0.963 | 0.852 | 16 | -1.130 | 0.275 |

**Supplementary Table 9.** DDIMER (log-scale) after 24, 72 and 168 compared to the baseline T0 of patients in VNS in comparison to SOC. *difference statistically significant

| **Parameter** | **Value** | **Std.Error** | **DF** | **t-value** | **p-value** |
| --- | --- | --- | --- | --- | --- |
| *(Intercept)* | 6.534 | 0.547 | 17 | 11.947 | <0.001 |
| *T24 vs. T0* | 0.543 | 0.403 | 17 | 1.347 | 0.196 |
| *T72 vs. T0* | 0.641 | 0.425 | 17 | 1.507 | 0.150 |
| *T72 vs. T0* | 0.581 | 0.417 | 17 | 1.392 | 0.182 |
| *Group VNS vs. SOC* | 0.187 | 0.773 | 8 | 0.241 | 0.815 |
| *T24 vs. T0 x Group* | -0.550 | 0.554 | 17 | -0.993 | 0.334 |
| *T72 vs. T0 x Group* | -0.577 | 0.586 | 17 | -0.986 | 0.338 |
| *T72 vs. T0 x Group* | -0.643 | 0.574 | 17 | -1.121 | 0.278 |

**Supplementary Table 10.** Ferritin (log-scale) after 24, 72 and 168 compared to the baseline T0 of patients in VNS in comparison to SOC. *difference statistically significant

| **Parameter** | **Value** | **Std.Error** | **DF** | **t-value** | **p-value** |
| --- | --- | --- | --- | --- | --- |
| *T24 vs. T0* | 1.478 | 0.110 | 17 | 13.436 | <0.001 |
| *T72 vs. T0* | -0.120 | 0.089 | 17 | -1.360 | 0.192 |
| *T72 vs. T0* | -0.055 | 0.115 | 17 | -0.479 | 0.638 |
| *Group VNS vs. SOC* | 0.480 | 0.140 | 17 | 3.435 | 0.003 |
| *T24 vs. T0 x Group* | 0.010 | 0.156 | 8 | 0.061 | 0.953 |
| *T72 vs. T0 x Group* | 0.027 | 0.120 | 17 | 0.228 | 0.823 |
| *T72 vs. T0 x Group* | -0.196 | 0.161 | 17 | -1.221 | 0.239 |
| *T24 vs. T0* | **-0.723** | **0.197** | **17** | **-3.677** | **0.002*** |

**Supplementary Table 11.** Ferritin (log-scale) after 24, 72 and 168 compared to the baseline T0 of patients in VNS in comparison to SOC. *difference statistically significant
